# IL-12 protects from psoriasiform skin inflammation

**DOI:** 10.1038/ncomms13466

**Published:** 2016-11-28

**Authors:** Paulina Kulig, Stephanie Musiol, Sandra Nicole Freiberger, Bettina Schreiner, Gabor Gyülveszi, Giancarlo Russo, Stanislav Pantelyushin, Kenji Kishihara, Francesca Alessandrini, Thomas Kündig, Federica Sallusto, Günther F.L. Hofbauer, Stefan Haak, Burkhard Becher

**Affiliations:** 1 Institute of Experimental Immunology, University of Zurich, 8057 Zurich, Switzerland; 2 Experimental Immunology Unit, Centre of Allergy and Environment (ZAUM), Technical University of Munich and Helmholtz Centre Munich, 80802 Munich, Germany; 3 Department of Dermatology, University Hospital Zurich, 8091 Zurich, Switzerland; 4 Institute for Research in Biomedicine, Cellular Immunology, 6500 Bellinzona, Switzerland; 5 Functional Genomics Center Zurich, University of Zurich and ETH Zurich, 8057 Zurich, Switzerland; 6 Department of Immunology, Faculty of Pharmaceutical Sciences, Nagasaki International University, 859-3298 Nagasaki, Japan

## Abstract

Neutralization of the common p40-subunit of IL-12/23 in psoriasis patients has led to a breakthrough in the management of moderate to severe disease. Aside from neutralizing IL-23, which is thought to be responsible for the curative effect, anti-p40 therapy also interferes with IL-12 signalling and type 1 immunity. Here we dissect the individual contribution of these two cytokines to the formation of psoriatic lesions and understand the effect of therapeutic co-targeting of IL-12 and IL-23 in psoriasis. Using a preclinical model for psoriatic plaque formation we show that IL-12, in contrast to IL-23, has a regulatory function by restraining the invasion of an IL-17-committed γδT (γδT17) cell subset. We discover that IL-12 receptor signalling in keratinocytes initiates a protective transcriptional programme that limits skin inflammation, suggesting that collateral targeting of IL-12 by anti-p40 monoclonal antibodies is counterproductive in the therapy of psoriasis.

## Abstract (web summary)

IL-12 and IL-23 share the common p40 subunit yet have distinct immunological functions with IL-12 typically contributing to Th1 responses and IL-23 to Th17 responses. Here the authors show that current p40 based therapies for psoriasis are counterproductive owing to an IFN-γ-independent tissue protective function of IL-12 in skin.

## Graphical abstract

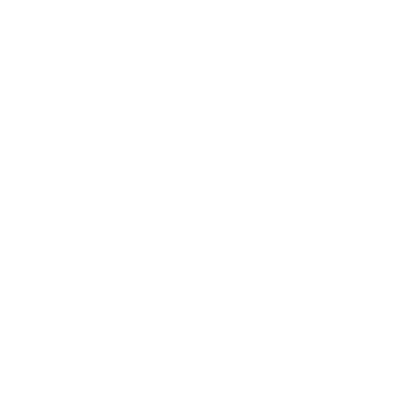

Psoriasis is a chronic relapsing-remitting inflammatory skin disease that develops in genetically predisposed individuals after an unknown initial environmental, pathogenic or internal trigger. It is characterized by thickened epidermis (acanthosis), a result of keratinocyte hyper-proliferation, dysregulated keratinocyte differentiation (for example, parakeratosis), increased vascularity and accumulation of inflammatory infiltrates of T cells, neutrophils and dendritic cells^1^. As T helper (T_H_)-17, T_H_22 and T_H_1 cells are found in psoriatic lesions^2^, current understanding of the disease pathogenicity proposes a model in which activated dendritic cells and macrophages express IL-12 and IL-23, which polarize autoreactive T cells into their subsequent effector phenotype^3^. As IL-12 and IL-23 are detected in psoriatic lesions^4^,^5^, targeting both cytokines concomitantly (through neutralization of the common IL-12/23p40 subunit) may have a synergistic therapeutic effect. Indeed, two anti-IL-12/23p40 monoclonal antibodies (mAbs; ustekinumab and briakinumab) have been effective in the treatment of psoriasis vulgaris, and ustekinumab is now registered for clinical use^6^,^7^,^8^,^9^.

However, data from mouse models as well as clinical studies demonstrates the IL-23/IL-17 axis to be the dominant pathway in the pathogenesis of the disease^10^. Repeated intradermal injections of IL-23 in mice led to development of a psoriasiform inflammatory phenotype^11^, and IL-23-driven effector cytokines, IL-17A, IL-17F and IL-22, have been described as important factors in psoriatic plaque formation^12^,^13^. Moreover, genome-wide associated studies point at several genes of the IL-23 pathway, such as *IL23R*, *IL12b and IL23a*, as risk factors for psoriasis^14^. Although T_H_17 cells are implicated as effector cells in psoriasis, data generated in the Aldara-induced psoriatic plaque formation model show that psoriatic lesion development can be independent of αβT cells, but relies on the activity of a specific subset of γδT17 cells discriminated by their Vγ4^+^ T-cell receptor (TCR) (nomenclature according to Heilig and Tonegawa)^15^. In concert with type 3 innate lymphoid cells, γδT cells are the major source of IL-17A, IL-17F and IL-22 in the inflamed skin^16^. Innate-like lymphocytes constitutively express high levels of the IL-23 receptor^12^,^17^, making them immediate responders to IL-23 and therefore suggesting a potential role in lesion formation. IL-17A-neutralizing antibodies ixekizumab and secukinumab, as well as an IL-17A receptor-blocking antibody, brodalumab, have successfully been tested in psoriasis patients^18^,^19^,^20^. First published results showed strong clinical improvement and strengthen the notion that the IL-23/IL-17 axis is essential in the pathogenesis of psoriasis. Moreover, a clinical study on anti-IL-23p19 mAb (guselkumab) confirmed a pathogenic role for de-regulated IL-23 in psoriasis^21^.

Whereas IL-23p19 and IL-23p40 transcripts have been shown to be increased in psoriatic lesions, IL-12p35 was not^22^. IL-12 and IL-23 are structurally related and mainly expressed by activated dendritic cells and macrophages. Despite their similarities, both cytokines trigger vastly divergent immunological pathways. IL-12 is an important factor for the differentiation of naive T cells into interferon-γ (IFN-γ)-producing T_H_1 cells, whereas IL-23 plays a role in sustenance of T_H_17 responses^23^,^24^. It has been shown that neutralization of IL-12 leads to the amelioration of psoriasis-like skin disorder in mice. However, the antibody used was targeting the common p40 subunit, thus neutralizing both IL-12 and IL-23 (ref. 25). The therapeutic activity of the p40-specific antibody is possibly due to the inhibition of IL-23 signalling pathway and not IL-12 (ref. 26). Nonetheless, the presence of IL-12 and IL-12-induced factors has been reported in human psoriatic lesions, which suggests their potential involvement in disease pathology^27^,^28^. The collateral inhibition of IL-12 and its potential affect on psoriasis is currently under discussion, but the actual contribution of IL-12 to the formation of psoriasiform lesions has never been addressed. Clinical studies suggest a superior efficacy through neutralization of the IL-23/IL-17 axis over blockade of IL-12/23p40 (refs 29, 30, 31). The aim of this study is to compare the individual roles of IL-12 and IL-23 in the pathophysiology of psoriatic plaque formation *in vivo*. We show that IL-12 opposes the pathogenic function of IL-23 in psoriatic plaque formation by signalling to the local stroma thereby restricting the cellular niche for type 17 T-cell accumulation. Our findings correct the prevailing view that IL-12 serves a primarily pro-psoriatic function during psoriatic plaque formation.

## Results

### Divergent roles of IL-12 and IL-23 in psoriatic inflammation

**Figure 1:**
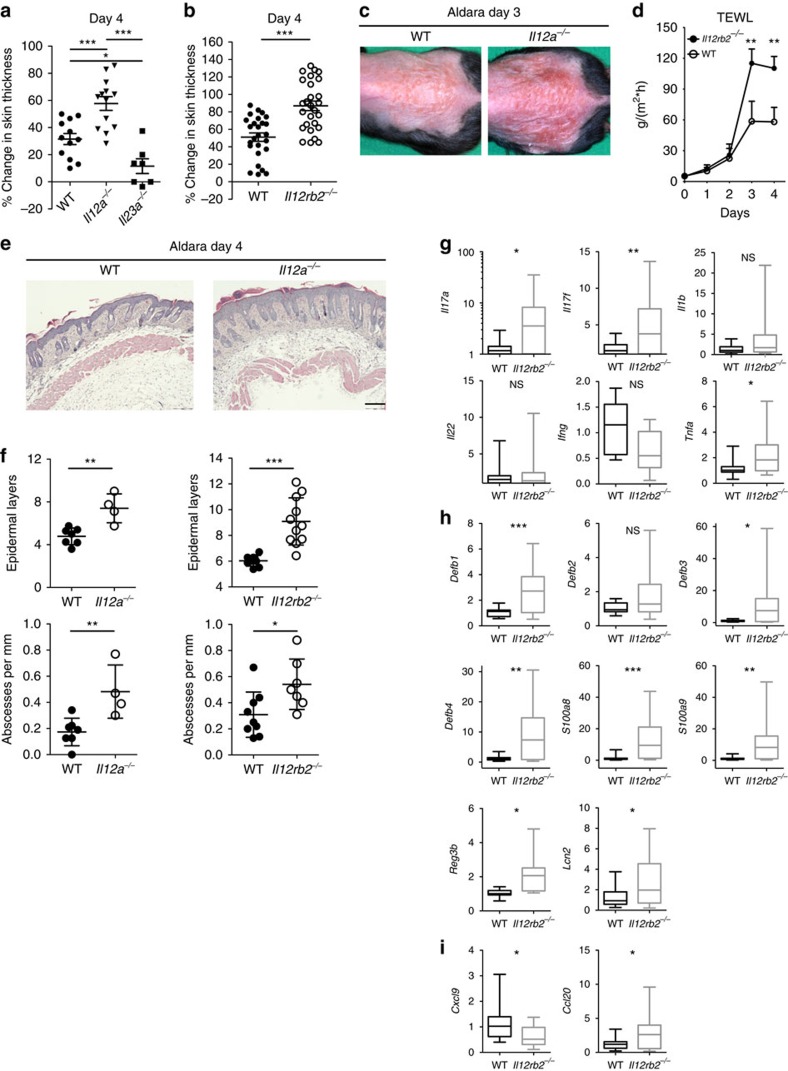
Psoriatic plaque formation in Aldara-treated IL-12- and IL-23-deficient mice. (**a**,**b**) WT, *Il12a*^*−/−*^, *Il23a*^*−/−*^ and *Il12rb2*^*−/−*^ mice were treated with Aldara for 6 days. Back skin lesions during peak disease (day 4) represented as per cent change in skin thickness compared with untreated skin on day 0. Cumulative representation of (**a**) four independent experiments, (*n*=12 per WT, 13 per *Il12a*^*−/−*^ and 7 per *Il23a*^*−/−*^, average mean±s.e.m.) and (**b**) nine independent experiments (*n*=26 per WT and 27 per *Il12rb2*^*−/−*^, average mean±s.e.m.). (**c**) Representative photos taken on day 3 post Aldara treatment. (**d**) Measurement of TEWL in Aldara-treated back skin. Cumulative graph of four independent experiments (*n*=9 per WT and 10 per *Il12rb2*^*−/−*^, average mean±s.e.m.). (**e**) Back skin sections stained with haematoxylin and eosin on day 4 post treatment; scale bar, 200 μm. (**f**) Quantification of mouse skin histology: total counts of epidermal layers and skin abscesses. Cumulative graph of 2–3 independent experiments (*n*=7–9 per WT, 4 per *Il12a*^*−/−*^ and 7–11 per *Il12rb2*^*−/−*^, average mean±s.e.m.). (**g**) Real-time quantitative PCR analysis of the whole skin on day 5 post treatment. Cumulative graphs of three independent experiments representing fold changes relative to the average WT expression levels of independent experiments (*n*=10–18 per WT and 10–15 per *Il12rb2*^*−/−*^). Data shown as box plots visualizing the distribution by min and max (whiskers) the 25^th^–75^th^ percentile (box) and median (band). Each data point represents an individual mouse. *P<0.05, **P<0.01, ***P<0.001 (**a**,**b**) one way analysis of variance (ANOVA) with Bonferroni post test, (**d**) two-way ANOVA with Bonferroni post test, (**f**–**i**) unpaired two-tailed *t*-test). NS, not significant.

Equally to the human disease, psoriatic lesions induced by topical Aldara treatment of mice can be reduced by application of neutralizing anti-p40 mAbs^16^. Psoriatic plaque formation is also greatly decreased in mice deficient in p40 (*Il12b*^*−/−*^) (Supplementary Fig. 1). Correspondingly, critical markers of psoriasiform inflammation in this model, such as neutrophil invasion and the accumulation of IL-17A-secreting Vγ4^+^ γδT cells in the lesions were reduced (Supplementary Fig. 2). To clarify the individual contributions of IL-12 and IL-23 to plaque formation we monitored the clinical disease course in mice deficient in the subunit specific to IL-12 (p35, *Il12a*^*−/−*^), its receptor (*Il12rb2*^*−/−*^) and IL-23 (p19, *Il23a*^*−/−*^). Whereas IL-12 is critical for type 1 immune responses, which in turn are thought to contribute to plaque formation, *Il12a*^*−/−*^ mice (unaltered IL-23 signalling) developed significantly more severe inflammation compared with wild-type (WT) mice (Fig. 1a). Mice lacking the IL-12-specific receptor subunit (*Il12rb2*^*−/−*^), likewise, developed more severe lesions (Fig. 1b and Supplementary Fig. 1), pointing towards a regulatory role of IL-12 in psoriasiform lesion formation. Of note, when we compared the skin pathology of IL-23-deficient mice, *Il12b*^*−*/*−*^ (lacking IL-12 and IL-23) and *Il23a*^*−*/*−*^ (only lacking IL-23), we also observed a trend towards a protective effect of IL-12 (Supplementary Fig. 1).

The aggravated psoriatic plaque formation in mice defective in IL-12 signalling encompassed accelerated disease progression as well as more severe scaling and erythema, which suggested a compromised skin barrier function (Fig. 1c). For quantification of barrier integrity we measured trans-epithelial water loss (TEWL)^32^ confirming that in the absence of IL-12 signalling Aldara treatment resulted in a more pronounced breach of epithelial barrier (Fig. 1d). Histopathologic features were also more pronounced, and we observed increased frequencies of micro-abscesses, mostly consisting of neutrophils in the stratum corneum, and increased acanthosis, when IL-12 was absent (Fig. 1c,e,f). Cytofluorometric quantification validated the amplified recruitment of neutrophils into the skin of *Il12a*^*−/−*^ mice (Supplementary Fig. 3). The data collectively suggest that collateral targeting of IL-12 signalling in psoriasis could impede the therapeutic efficacy of targeting IL-23.

The prototype function of IL-12 is to induce type 1 responses and to determine whether the regulatory effect of IL-12 in plaque formation is mediated through IFN-γ, we induced Aldara lesions in *Ifng*^*−/−*^ mice (Supplementary Fig. 4). In contrast to *Il12a*^*−/−*^ mice, *Ifng*^*−/−*^ mice had a slightly less severe course of disease. This demonstrates two points: (a) the protective role of IL-12 works independent of IFN-γ; and (b) IFN-γ itself is pro-inflammatory and promotes plaque formation.

To understand the molecular processes involved in the exaggeration of the psoriatic inflammatory response in IL-12 signalling-deficient compared with WT mice, transcript analysis of the respective lesional skin was performed (Fig. 1g–i). Hallmark pathways of psoriasis were analysed and among the cytokines IL-17A and IL-17F were elevated in the absence of IL-12Rβ2 (Fig. 1g). As expected, the inflamed skin of *Il12rb2*^*−/−*^ mice also showed a marked decrease of IFN-γ. We thus interrogated downstream transcripts relevant to psoriasiform inflammation induced by type 17 cytokines and found a range of antimicrobial peptides to be significantly upregulated (Fig. 1h). At the same time lipocalin 2 (*Lcn2*) revealed increased neutrophil activity in *Il12rb2*^*−/−*^ lesions, also indicative of an increased type 17 bias. Amongst relevant chemokines, CXCL9, which is dominantly controlled by IFN-γ, was found to be decreased (Fig. 1i). We found CCL20 to be significantly increased in the lesion of *Il12rb2*^*−/−*^ mice (Fig. 1i). CCL20 is the ligand of CCR6, a marker of dermato-tropic type 17 effector T cells, like γδT17 and T_H_17 cells.

### IL-12 controls invasion of Vγ6Vδ1^+^ γδT17 cells into the skin

**Figure 2:**
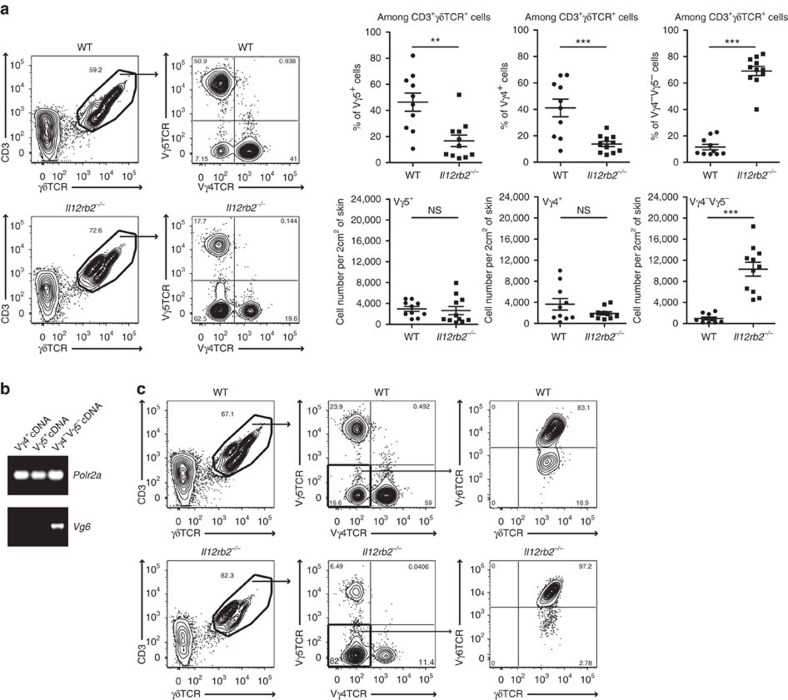
γδT cell distribution in Aldara-treated skin of WT and *Il12rb2*^*−/−*^ animals. (**a**) Representative plots and cumulative graphs of three independent experiments depicting flow cytometry analysis of inflamed skin; cells were gated on CD45^+^CD11b^*−*^ leukocytes and analysed for the presence of skin-resident and skin-infiltrating γδT cells (*n*=10 per WT and 11 per *Il12rb2*^*−/−*^, average mean±s.e.m.); please find Supplementary Fig. 5 depicting representative flow cytometry gating strategy. (**b**) Vγ4^+^, Vγ5^+^ and Vγ4^*−*^Vγ5^*−*^ γδT cells were sorted from *Il12a*^*−/−*^ Aldara-treated skin, and real-time quantitative PCR analysis for *Vg6* expression was performed. *Polr2a* was used as a house-keeping gene. (**c**) Representative contour plot of skin-infiltrating Vγ6^+^ γδT cells on Aldara treatment. Each data point represents an individual mouse. **P<0.01, ***P<0.001 ((**a**) unpaired two-tailed *t*-test). NS, not significant.

**Figure 3:**
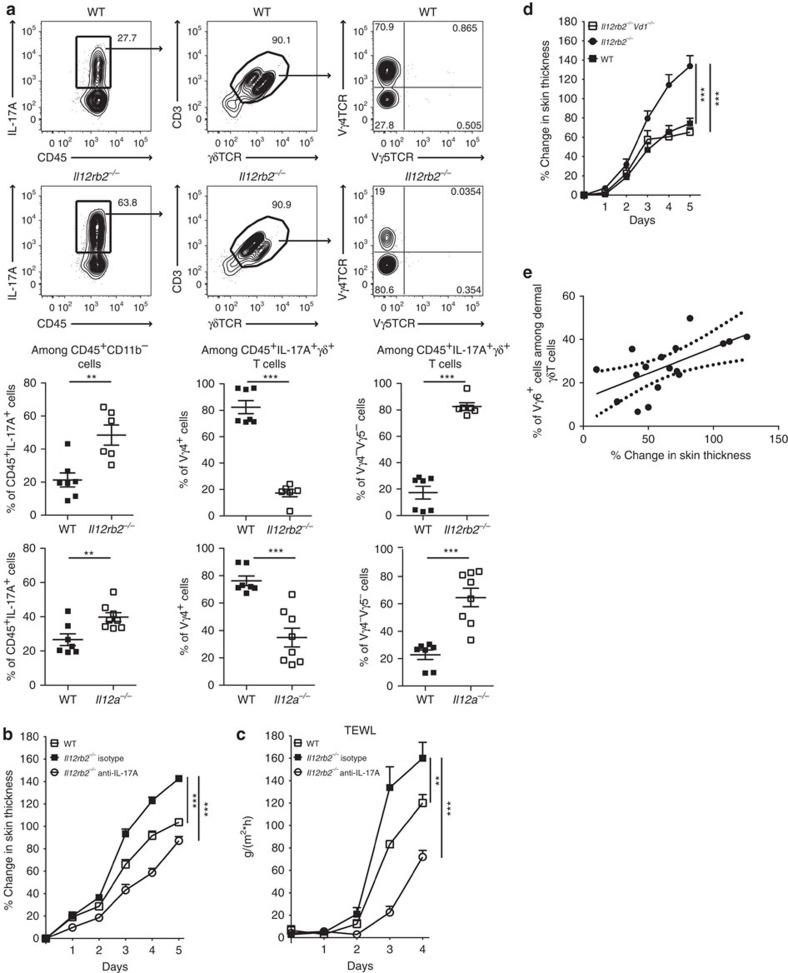
Vγ6^+^ γδT17 exacerbate psoriasiform inflammation. WT, *Il12a*^*−/−*^, *Il12rb2*^*−/−*^ and *Il12rb2*^*−/−*^*Vd1*^*−/−*^ mice were treated with Aldara for 6 days. (**a**) Representative plots and cumulative graphs of two independent experiments depicting flow cytometry analysis of inflamed skin; cells were gated on CD45^+^CD11b^*−*^ leukocytes and analysed for the expression of IL-17A (*n*=7 per WT, 6 per *Il12rb2*^*−/−*^ and 8 per *Il12a*^*−/−*^, average mean±s.e.m.). (**b**,**c**) Representative experiment out of three depicting psoriatic plaque formation in animals treated with neutralizing antibody against IL-17A. (**b**) Change in skin thickness compared with untreated skin on day 0 (*n*=3 per WT, 4 per *Il12rb2*^*−/−*^isotype and 5 per *Il12rb2*^*−/−*^ anti-IL-17A, average mean±s.e.m.) and (**c**) measurement of TEWL in Aldara-treated back skin (*n*=3 per WT, 4 per *Il12rb2*^*−/−*^ isotype and 5 per *Il12rb2*^*−/−*^ anti-IL-17A, average mean±s.e.m.). (**d**) Back plaque formation represented as per cent change in skin thickness compared with untreated skin on day 0. Cumulative graph of five independent experiments (*n*=17 per WT, 15 per *Il12rb2*^*−/−*^ and 12 per *Il12rb2*^*−/−*^*Vd1*^*−/−*^, average mean±s.e.m.). (**e**) Pearson's correlation analysis of Vγ6^+^ γδT cells in inflamed back skin calculated as per cent of all dermal γδT cells and per cent change in epidermal thickening in WT mice (*n*=17). Each data point represents an individual mouse. **P<0.01, ***P<0.001 ((**a**) unpaired two-tailed *t*-test, (**b**–**d**) two-way ANOVA with Bonferroni post test).

IL-17A-producing Vγ4^+^ γδT cells are the main drivers of the psoriasiform inflammatory processes in the skin and an established marker for disease severity^16^. However, the skin of Aldara-treated *Il12rb2*^*−/−*^ mice had a distinct decrease in the frequency of these cells (Fig. 2a and Supplementary Fig. 5). The increase in total γδT cell infiltration was attained by the appearance of another γδT cell subpopulation. Those cells neither expressed the Vγ4 (characteristic for highly mobile IL-17A-secreting, skin-invading T cells) nor the Vγ5 chain of skin-resident dendritic epidermal T cells (DETCs). Whereas Vγ4^*−*^Vγ5^*−*^ γδT cells can be found in low numbers in psoriatic lesion of WT mice, they accumulated in the inflamed skin of mice deficient in IL-12Rβ2 (Fig. 2a). We identified the Vγ4^*−*^Vγ5^*−*^ γδT cell population as the invariant γδT cell subset expressing Vγ6Vδ1^+^ TCR chains (Fig. 2b,c). This finding corresponds with the recent report^33^, in which Vγ6^+^ T cells were described as an IL-17-secreting effector subset with skin-homing capacity. The overall levels of IL-17A in *Il12a*^*−/−*^ and *Il12rb2*^*−/−*^ mice were increased with Vγ6^+^ γδT cells being main producers of IL-17A (Fig. 3a). To determine whether the elevated levels of IL-17A are responsible for the exacerbated psoriasiform response of *Il12rb2*^*−/−*^ animals, we found that neutralization of IL-17A led to the reduction in skin thickness, improved epidermal integrity and reduced neutrophil invasion (Fig. 3b,c and Supplementary Fig. 6). Of note, the overall cell numbers of effector Vγ4 and Vγ6 γδT cells were not altered (Supplementary Fig. 6).

To address if Vγ6^+^ T cell accumulation in the skin is directly coupled to IL-12 deficiency or rather a consequence of the exaggerated inflammation in the mutant mice (*Il12rb2*^*−/−*^ and *Il12a*^*−/−*^), we went back to the Aldara-treated *Il12b* and *Il23a* deficient mice, which both lack IL-23 and only differ in their deficiency and sufficiency of IL-12, respectively. Interestingly, we noticed that even in mice lacking IL-23 and thus, full-blown inflammation, we observed differences in Vγ6^+^ T-cell accumulation suggesting that IL-12 regulates the γδT17 cells in the skin (Supplementary Fig. 7). To test if Vγ6^+^ γδT17 cell accumulation is causative in the aggravation of the disease when IL-12 is absent, we bred *Vd1*^*−/−*^ mice, which selectively lack functional Vγ6Vδ1^+^ T cells^34^,^35^, on to *Il12rb2*^*−/−*^ background. Lack of Vγ6Vδ1^+^ cells consistently reduced the exaggerated disease phenotype in IL-12R mutants (Fig. 3d), suggesting a pivotal role of the Vγ6^+^ γδT17 cell subset in pathogenesis under the control of IL-12. This hyper-pathogenic potential of Vγ6^+^ effector cells extends its biological relevance to non-mutant WT mice, as we noted a positive correlation between skin thickening—a robust clinical parameter of psoriatic plaque formation—and the ratio of Vγ6^+^ effectors amongst the pool of dermal γδT cells in WT mice (Pearson's *r*=0.615, *P*=0.0086; Fig. 3e).

**Figure 4:**
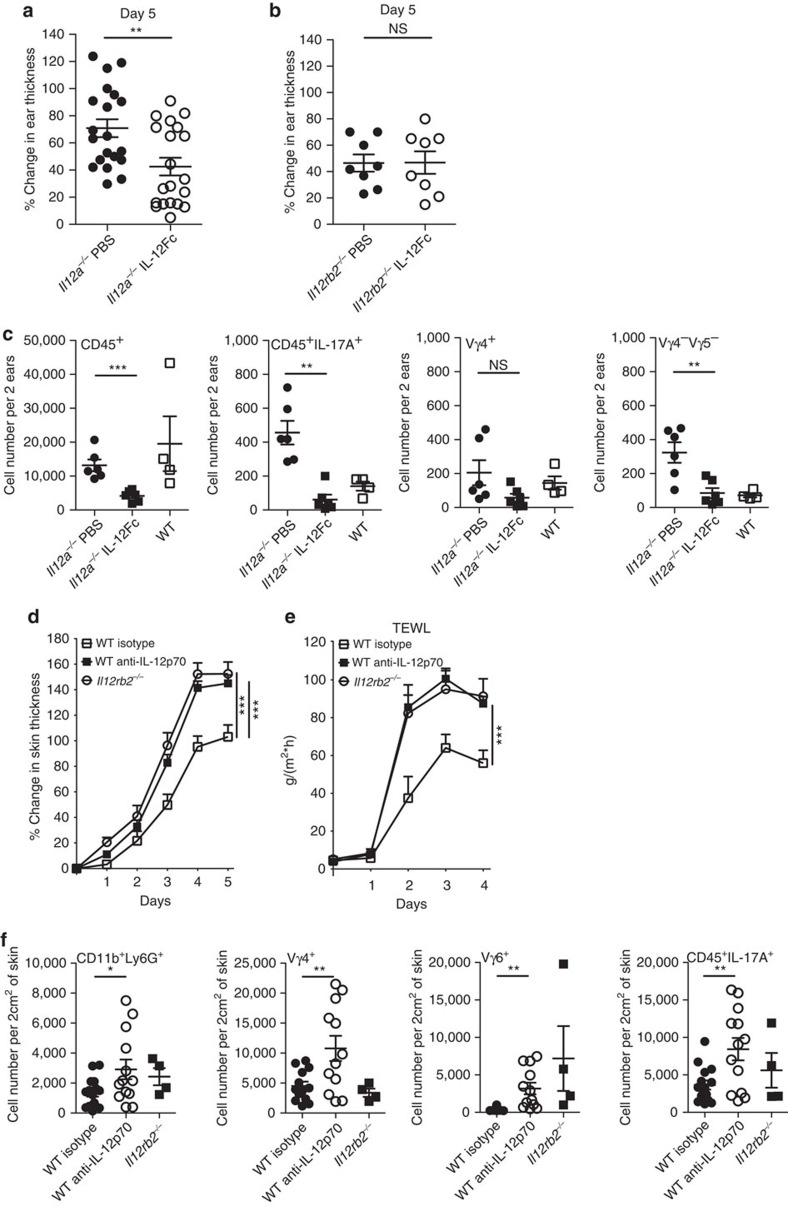
IL-12 limits skin inflammation. (**a**–**c**) WT, *Il12a*^*−/−*^ and *Il12rb2*^*−/−*^ mice were treated with Aldara, 200 ng of IL-12Fc or PBS was injected every second day starting on day −1. (**a**,**b**) Ear skin inflammation on day 5 represented as a per cent change in skin thickness compared with untreated skin on day −1. Cumulative graphs of (**a**) four and (**b**) two independent experiments (*n*=20 per *Il12a*^*−/−*^ and 8 per *Il12rb2*^*−/−*^, average mean±s.e.m.). (**c**) Flow cytometry analysis of inflamed back skin; absolute numbers of skin-infiltrating CD45^+^ leukocytes, Vγ4^*−*^Vγ5^*−*^ γδT, Vγ4^+^ γδT cells and CD45^+^IL-17A^+^ leukocytes. Cumulative graphs of three independent experiments (*n*=6 per *Il12a*^*−/−*^ and 4 per WT, average mean±s.e.m.). (**d**–**f**) WT and *Il12rb2*^*−/−*^ mice were treated with Aldara, 200 μg of anti-IL-12p70 antibody or isotype control was injected every second day starting on day −1. (**d**,**e**) Cumulative graphs of four independent experiments depicting (**d**) per cent change in skin thickness compared with untreated skin on day 0 (*n*=8 per WT isotype, 13 per WT anti-IL12p70 and 10 per *Il12rb2*^*−/−*^, average mean±s.e.m.) and (**e**) measurement of TEWL in Aldara-treated back skin (*n*=8 per WT isotype, 14 per WT anti-IL12p70 and 10 per *Il12rb2*^*−/−*^, average mean±s.e.m.). (**f**) Flow cytometry analysis of inflamed back skin; absolute numbers of skin-infiltrating neutrophils, Vγ4^+^ γδT cells, Vγ6^+^ γδT cells and CD45^+^IL-17A^+^ leukocytes. Cumulative graphs of three independent experiments (*n*=13 per WT isotype, 12 per WT anti-IL12p70 and 4 per *Il12rb2*^*−/−*^, average mean±s.e.m.). Each data point represents an individual (**a**,**b**) ear or (**c**,**f**) mouse. *P<0.05, **P<0.01, ***P<0.001 ((**a**–**c**,**e**,**f**) unpaired two-tailed *t*-test, (**d**) two-way ANOVA with Bonferroni post test). NS, not significant.

The p35 subunit is shared between two members of the IL-12 superfamily, IL-12 (p35/p40) and IL-35 (p35/EBI3). Although IL-35 has been demonstrated to have predominantly regulatory functions^36^, *Ebi3*^*−/−*^ mice did not phenocopy the aggravated pathology of IL-12 mutants, demonstrating that IL-12 and not IL-35 exerts protective functions in psoriasis (Supplementary Fig. 8). This was confirmed by local IL-12 administration, which reduced inflammation in *Il12a*^*−/−*^ mice (Fig. 4a) but not in *Il12rb2*^*−/−*^ mice (Fig. 4b). Correspondingly, IL-12 administration directly to the lesion diminished the invasion by γδT17 cells (Fig. 4c). We also treated WT mice with anti IL-12p70 heterodimer-specific neutralizing antibody, and observed increased skin thickness as well as decreased skin integrity (Fig. 4d,e). The absolute numbers of inflammatory infiltrates, including neutrophils, effector γδT cells and IL-17A-producing cells were also enhanced (Fig. 4f). The combination of these loss- and gain-of-function *in vivo* experiments led us to conclude that IL-12, which is produced within the inflammatory lesion, limits inflammation by restricting the numbers of pathogenic γδT17 cells.

### IL-12 elicits a protective programme in keratinocytes

**Figure 5:**
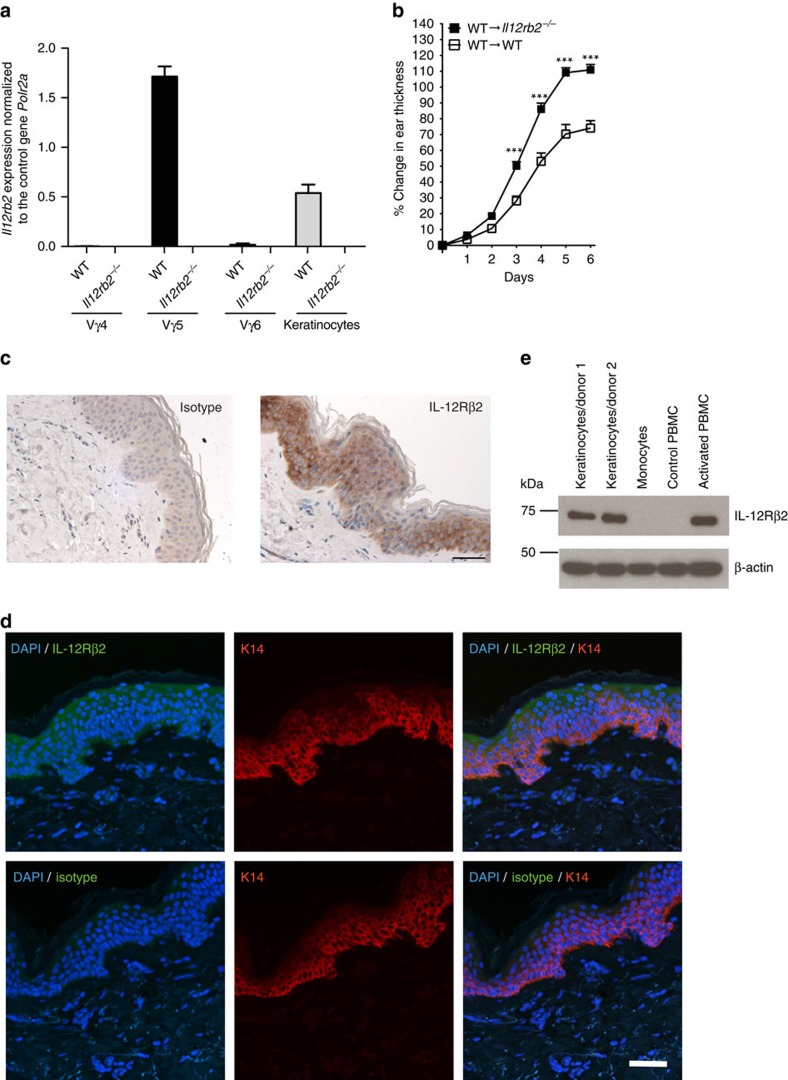
IL-12 responding cells in psoriatic skin. (**a**) WT and *Il12rb2*^*−/−*^ mice were treated with Aldara for 6 days. Vγ4^+^, Vγ5^+^ and Vγ6^+^ γδT cells were sorted by flow cytometry from WT and *Il12rb2*^*−/−*^ Aldara-treated skin, and real-time quantitative PCR analysis for *Il12rb2* expression was performed. *Polr2a* was used as a house-keeping gene. Cumulative graph of four independent experiments (*n*=4 per WT and *Il12rb2*^*−/−*^, average mean±s.e.m.). Keratinocytes were sorted from naive WT and *Il12rb2*^*−/−*^ animals, and *Il12rb2* expression analysis was performed. Cumulative graph of three independent experiments (*n*=3 per WT and *Il12rb2*^*−/−*^, average mean±s.e.m.). (**b**) Bone marrow chimeras were treated with Aldara for 7 days. Ear skin inflammation represented as a per cent change in ear thickness compared with untreated ear on day 0. Cumulative graph of four independent experiments (*n*=17 per WT into WT and 16 per WT into *Il12rb2*^*−/−*^, average mean±s.e.m.). (**c**,**d**) Skin sections from healthy human donors were stained with antibodies against (**c**) human IL-12Rβ2 or total rabbit IgG for immunohistochemistry; scale bar, 50 μm, and (**d**) human IL-12Rβ2 or total rabbit IgG (green), K14 (red) and 4,6-diamidino-2-phenylindole (DAPI; blue) for immunofluorescent staining; scale bar, 50 μm. (**e**) Immunoblot analysis of IL-12Rβ2 in human primary keratinocytes. Human monocytes, naive and activated PBMCs were used as negative and positive controls; please find Supplementary Fig. 11 showing uncropped western blot data. Each data point represents an individual (**a**) sort of pooled material of two mice (**b**) mouse. ***P<0.001 ((**b**) two-way ANOVA with Bonferroni post test).

The IL-12R complex is expressed on certain subsets of natural killer (NK) cells, NK T cells, γδT cells and activated αβT cells^37^. Here we show the regulation of γδT17 cell accumulation in the skin mediated by IL-12 signalling. Accordingly, we hypothesized a direct response of γδT17 cells to IL-12. For this, we determined *Il12rb2* expression directly on skin-associated γδT cells (Vγ4^+^, Vγ6^+^ and Vγ5^+^ subsets) sorted from psoriatic WT and *Il12rb2*^*−/−*^ skin. As expected, we found high levels of *Il12rb2* RNA in Vγ5^+^ DETCs. However, we failed to detect *Il12rb2* transcripts in the dermal Vγ4^+^ and Vγ6^+^ cell populations (Fig. 5a). IL-12 stimulation of DETC, similarly to T_H_1 effector cells, induces a type 1 cytokine responses dominated by IFN-γ (ref. 38), which however did not mediate a protective effect of IL-12 in psoriatic plaque formation (Supplementary Fig. 4). Furthermore, DETC have been excluded from contributing to psoriatic plaque formation by a comprehensive study of Stockinger and colleagues using aryl hydrocarbon receptor mutant mice^39^, based on the observation that cell intrinsic aryl hydrocarbon receptor deficiency in γδT cells leads to an almost complete absence of skin-resident DETCs while other dermal γδT cell populations are not affected^40^,^41^. As we did not find a possible direct link between IL-12 and its effect on γδT cells, we screened the skin for alternative IL-12 responders. Keratinocytes actively participate in the regulation of immune responses in the psoriatic skin^42^,^43^, and there are several reports demonstrating that keratinocytes are responsive to IL-12 (refs 44, 45, 46) and that IL-12R engagement activates both STAT-3 and STAT-4 in keratinocytes, which protects against ultraviolet-mediated skin damage^47^.

We thus sorted CD49f^high^ keratinocytes from naive WT and *Il12rb2*^*−/−*^ mice (Supplementary Fig. 9) and detected *Il12rb2* transcripts (Fig. 5a). To determine whether the IL-12R expression by keratinocytes would explain the exacerbated disease phenotype of IL-12R-deficient mice, we generated bone marrow chimeras by transferring WT (together with neonatal thymocytes to provide γδT17 cells)^17^ into either WT or *Il12rb2*^*−/−*^ recipients (Supplementary Fig. 10a). Aldara treatment resulted in increased skin inflammation when IL-12R was missing from the skin stroma (Fig. 5b). The majority of effector T cells present in the Aldara-treated skin were indeed of donor origin (Supplementary Fig. 10b). Again, we observed increased inflammation and leukocyte skin invasion by neutrophils and IL-17-producing T cells in the mice, where IL-12R was lacking in keratinocytes (Supplementary Fig. 10c).

These data open the possibility that in the context of psoriasiform lesion formation, not only lymphocytes but also the epidermal stroma has the capacity to directly respond to IL-12. To further elaborate on this important link and to translate our findings to the human skin, we examined clinical biopsies and found ample expression of IL-12Rβ2 protein in human epidermis (Fig. 5c). Confocal microscopy further confirmed IL-12Rβ2 co-localization with the keratinocyte marker K14 (Fig. 5d). To further ascertain the cellular identity of the IL-12Rβ2-bearing stroma, we expanded human primary keratinocytes *in vitro* and performed immunoblotting of the IL-12Rβ2 protein. Keratinocytes had abundant IL-12Rβ2 levels comparable to activated peripheral blood mononuclear cells (PBMCs), whereas no signal was detected in unstimulated human PBMCs or monocytes (Fig. 5e and Supplementary Fig. 11). Of note, we also found IL-12Rβ2 expression in human psoriatic skin (Supplementary Fig. 12).

**Figure 6:**
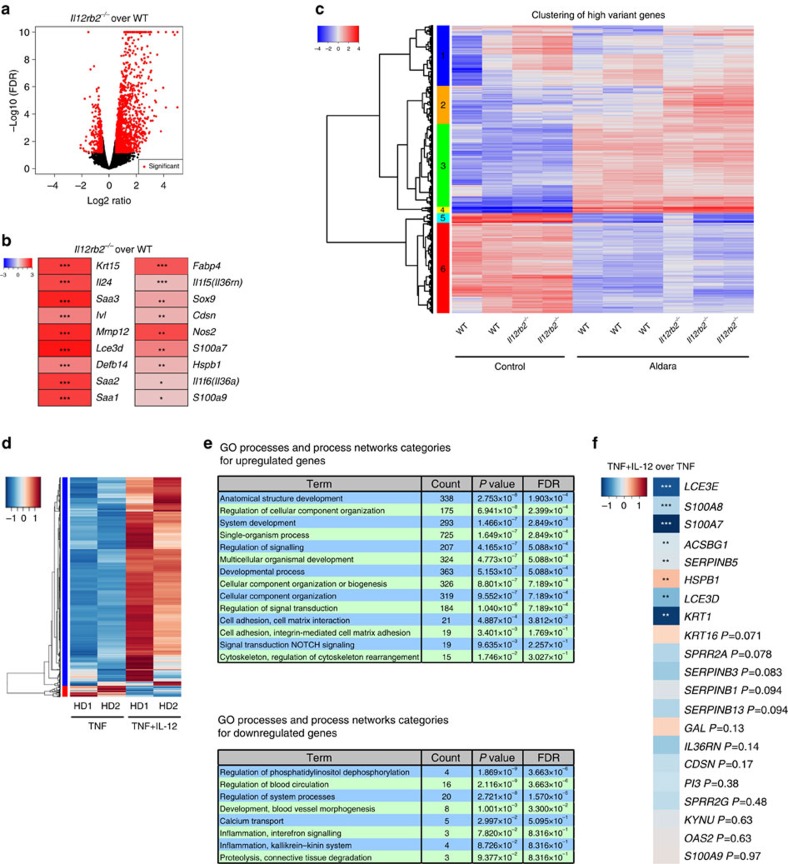
NGS of mouse and human keratinocytes. (**a**–**c**) NGS was performed on RNA extracted from mouse keratinocytes sorted from naive and Aldara-treated skin of WT and *Il12rb2*^*−/−*^ animals. (**a**) Volcano plot showing log2ratio versus –log10(FDR) (*Il12rb2*^*−/−*^ versus WT). (**b**) Heatmaps showing representative psoriasis-related genes regulated by IL-12 pathway. Significance is shown alongside. (**c**) Heatmap image of rank based top 2,000 genes with highest s.d. of log2 signal across samples. (**d**–**f**) NGS of human primary keratinocytes activated with TNF in the presence or absence of IL-12. (**d**) Differentially regulated transcripts between cells stimulated with TNF and IL-12 versus TNF stimulation alone are shown in a heatmap image for two individual healthy donors (HD; *n*=2). The blue colour represents low expression level while red indicates high expression levels. In all, 942 genes were significantly altered with *P*<0.05. (**e**) The enriched gene ontology and process network categories for up- and downregulated transcripts based on the differentially expressed genes. (**f**) Heatmap showing representative psoriasis-related genes regulated by IL-12 stimulation. Significance is shown alongside. *P<0.05, **P<0.01, ***P<0.001 ((**b**,**f**) unpaired two-tailed *t*-test). FDR, false discovery rate.

To gain unbiased insights into the molecular processes induced by IL-12 signalling in keratinocytes, we performed transcriptomic analysis by next-generation sequencing (NGS) of sorted keratinocytes from Aldara-treated WT and *Il12rb2*^*−/−*^ animals, at a time point before the mice display differential disease development. We identified >1,000 significantly altered expression features between the groups, conforming to a stringent significance threshold (*P*<0.001; Fig. 6a). Amongst the top 100 most significant differences between *Il12rb2*^*−/−*^(Aldara) and WT (Aldara), we found a broad range of factors closely associated with human psoriasis^48^,^49^ as well as mouse models of the disease, for example, involucrin (*Ivl*), late cornified envelope 3D (*Lce3d*), β14 defensin (*Defb14*), matrix metallopeptidase 12 (*Mmp12*), IL-24 (*Il24*), serum amyloid A (*Saa3*), transforming growth factor β2 (*Tgfb2*) and different serin peptidase inhibitors (Serpin). Figure 6b summarizes an excerpt of factors typically associated with psoriasis overlapping with those detected here.

The majority of transcriptional changes found in the comparison *Il12rb2*^*−/−*^(Aldara) versus WT(Aldara) were in gene families typically involved in tissue structure (Supplementary Fig. 13a), keratinocyte differentiation and basement membrane integrity (Supplementary Fig. 13b). Direct immune-related changes were smaller in number but highly significant. Besides inflammatory pathways generally active in skin inflammation like arachidonic acid metabolism and transforming growth factor-β an over-representation of inflammatory elements of the IL-17 tissue response (granulocyte colony-stimulating factor, antimicrobial peptides, IL-19/24 and IL-6) as well as a response modulator (IL-17RD) enhancing neutrophil engagement were found enhanced in *Il12rb2*^*−/−*^ lesions (Supplementary Fig. 13c).

We next analysed the top 10% genes selected on high variance between the Aldara-treated groups (WT versus *Il12rb2*^*−/−*^) and performed cluster- followed by Metacore analysis to determine the functional alterations elicited by IL-12 signalling (Fig. 6c and Supplementary Fig. 14). The result can be classified into three categories: transcripts (a) downregulated (clusters 5 and 6); (b) enhanced uniformly between the genetic groups (cluster 3); and (c) differentially regulated between the genetic groups (clusters 1, 2 and 4). Genes related to general cell metabolism and cell cycle (cluster 3) were hardly affected by IL-12 signalling in keratinocytes. Processes related to type 17 inflammation are drastically enhanced in the absence of IL-12 receptor (enriched in cluster 4; Supplementary Fig. 14), which is in agreement with the increased disease severity observed in these animals. Also, cell trafficking as well as tissue structure and remodelling (clusters 1 and 2; Supplementary Fig. 14) are affected. Unique to cluster 2 is that changes are confined to *Il12rb2*^*−*/*−*^ keratinocytes (Fig. 6c). This is of interest since the affected pathways/processes could be involved in opening and closing a cellular niche for γδT17 cell accumulation and consequentially elevated levels IL-17A within the psoriatic lesion.

We next tested if IL-12 elicits a response in primary human keratinocytes. As described previously, IL-12 alone does not significantly change the transcriptional profile of epidermal keratinocytes^47^, hence we pre-activated them to simulate the inflammatory context *in vitro*. We found 942 genes to be differentially expressed when IL-12 was applied to keratinocytes pre-activated with TNF (*P*<0.05, log2FC>0.5 and<−0.5). A response to IL-12 was consistently found across donors (Fig. 6d). Enrichment analysis for biological processes regulated by IL-12 revealed several processes such as multicellular organismal development, system development or cytoskeleton rearrangement to be most prominently enhanced, which are processes typical downregulated in human psoriatic skin compared with healthy^49^ (Fig. 6e). Interestingly, the processes found in this human Metacore analysis were similar to the ones found in clusters 2 and 4 of the mouse transcriptome (Supplementary Fig. 14), indicating that IL-12, indeed, could counter-regulate changes induced in the epidermal stroma inflicted by inflammatory stimuli.

To permit a deeper statistical analysis, we performed RNAseq on three samples of primary human keratinocytes per group (TNF versus TNF+IL-12) stimulated independently. It has been previously shown that *in vitro* stimulation of human keratinocytes mimics the transcriptional profile of the psoriatic epidermal stroma to some extent^50^, however, the changes induced by IL-12 in this *in vitro* model were not comparable in strength to the *ex vivo* analysis of keratinocytes in the psoriatic lesion of the mouse model. Despite this limitation there was a clear counter-modulation of the psoriasiform transcriptional pattern induced by TNF (refs 49, 50; Fig. 6f).

## Discussion

Treatment of most chronic inflammatory diseases used to imply broad immunosuppression. For the treatment of plaque-like psoriasis this has categorically changed in the last 10 years, as we are currently experiencing a rapid evolution of cytokine-blocking drugs. The initial milestone achievement was blocking of TNF, set-up to counter a number of different auto-inflammatory disorders, psoriasis being one of them^51^. Whilst effective, drug-induced TNF depletion retains to some extend the disadvantage of former approaches of a generalized immunosuppression, as well as a quota of one-third of eventual non-responders. A critical step towards better therapeutic specificity was led by early preclinical data showing that targeting of IL-12p40 successfully prevents or curbs pathology in numerous models of chronic inflammatory disease, which resulted in the development of neutralizing mAbs for clinical application^25^,^52^. Shortly after the discovery of IL-23 in 2003, however, it became apparent that the anti-p40 therapeutic approach inadvertently counteracted two major inflammatory pathways in parallel, IL-12 and IL-23. The predominant role of IL-23 in the pathogenesis of some chronic inflammatory disease, which was formerly claimed by IL-12, was first discovered in experimental autoimmune encephalomyelitis (EAE) a disease model for multiple sclerosis^53^,^54^,^55^. Nonetheless, anti-p40 mAb therapy performed with unprecedented efficacy in treatment of plaque-like psoriasis for which it became FDA approved in 2009 (ustekinumab). For the treatment of psoriasis there is an on-going effort to increase the focus on the blockade of the type 17 inflammatory response, for example, by inhibition of IL-23p19 (refs 21, 56) or IL-17 (refs 18, 19, 20), which in recent clinical trials exhibiting even higher efficacy than ustekinumab^30^. As predicted from the findings in preclinical models, all clinical data available to date point towards a particularly monomorphic patho-mechanism behind psoriatic plaque formation dominated by an unrestrained IL-23/17 effector response in the skin. Consequentially, it seems that blocking IL-23 or even single IL-23 downstream effector molecules should be sufficient to effectively treat psoriatic lesions.

However, at present, anti-p40, inhibiting IL-12 and IL-23 signalling, is applied as standard care (ustekinumab) for moderate to severe psoriasis vulgaris in adults. Therapeutic blocking of IL-12/23p40 is well studied, and corresponding indications have been monitored in the clinical studies of the two anti-p40 mAbs, ustekinumab and briakinumab^57^,^58^,^59^. Taken the long-term treatment perspective of those patients and in general the delicate line between beneficial immune-modulation and detrimental immunosuppression it is imperative to delineate all aspects of the drugs' biological effect. One concern of particular importance, considering the continuous treatment regime in psoriasis, is the prominent role of IL-12 in tumour control^60^,^61^. Indeed, briakinumab, although very effective and overall positively evaluated, showed a conspicuously increased rate of cancer in different clinical studies^6^,^62^. So far, it was hypothesized that blocking of IL-12 could extend the drug's anti-inflammatory effectiveness, as presence of T_H_1 cells and IFN-γ in psoriatic lesions has been reported^27^. But data on a causal relationship of either IL-12 or IFN-γ and psoriasis was—until now—missing. At the same time data presented from past and on-going comparative clinical studies of mAbs targeting solely the IL-23/IL-17 axis versus the share subunit of IL-23 and IL-12 attested a higher efficacy to the formed approach^29^,^30^, which is in conflict with the notion of a dominant pro-psoriatic role of IL-12 in the skin. Concordant with the correlative data from patient skin biopsies we found IFN-γ to be produced within the Aldara-induced psoriatic lesions (data not shown) and confirmed the pro-psoriatic function of IFN-γ. Here we found IL-12, which generally drives type 1 immune responses, to not only act independent of IFN-γ, but instead to suppress inflammation in the skin. Although surprising at first sight, a protective role of IL-12 in an inflammatory condition has been observed in other tissues, for example, in organ-specific autoimmune inflammation of the central nervous system. There, likewise to the clinical findings in our psoriasis model, IL-12 restrains part of the inflammatory response^53^,^54^. Also in EAE a dual role of IL-12 is debated, as type 1 immunity is partially considered to be instrumental in disease progression and central nervous system pathology, but on the other hand the net effect of IL-12 is clearly anti-inflammatory. The mechanistic underpinning of this in EAE, however, remains largely unclear.

It is only in the past few years that the significant contribution of γδT cells in type 17-driven diseases has been explored. Also in psoriatic lesions their contribution has been noted^12^,^63^,^64^,^65^, but the precise nature of their action in human inflammatory disease, as well as the translation of functional γδT cell subset identities remains unclear. Invariant γδT cells, like any innate lymphoid cell type, are low in frequency and their analysis in humans is hampered by the typically small sizes of tissue biopsies. With the data available to date it is hard to predict whether a homologue or functional orthologue of Vγ6^+^ γδT17 cells exists in humans and if accumulation of such cells is affected by IL-12. In mice, the presence of IL-12 prevents the unbridled accumulation of Vγ6^+^ type 17 cells, independent of Vγ4^+^ γδT17 cells, resulting in a net surplus of IL-17. Importantly, IL-12 did not affect skin-invading γδT17 cells directly.

Whereas TNF or IL-1β have long been recognized to interact with both haematopoietic and stromal cells, here we describe that IL-12 too can directly communicate with the stromal microenvironment, independent of its function in immune cells. In contrast to mediators such as TNF or IL-1β, which exacerbate epithelial inflammation^66^, IL-12 initiates a tissue-protective response in keratinocytes. Moreover, IL-12 counter-regulated the psoriatic transcriptional signature in both murine and human keratinocytes. IL-12 specifically modifies transcriptional programmes affecting tissue structure remodelling, which in turn could affect immune cell accumulation and recruitment allowing available pathogenic lymphocytes to populate the tissue. In the Aldara-induced psoriasis model, the cells accumulating in IL-12-deficient tissue are predominantly Vγ6^+^ type 17 cells. The increase in net IL-17 activates the local stroma (for example, keratinocytes) amplifying the type 17 signature in the tissue, which can explain the drastic increase in neutrophil influx and epidermal micro-abscesses in *Il12a*^*−/−*^ mice.

Thus, while our data causally relate psoriasis to be a bona fide type 17 inflammatory disorder with minor contribution of type 1 effector response, we additionally demonstrate that IL-12 mediates an autonomous regulatory programme in the skin. Whether these preclinical findings can be fully translated to human patients remains to be established, however, it does offer a lead towards understanding the higher clinical efficacy of anti-p19 and anti-IL-17 drugs compared with ustekinumab (anti-p40) in psoriasis vulgaris patients. The observed anti-psoriatic effect of IL-12 may not be exclusively mediated through its impact on the keratinocyte compartment, and additional IL-12-responsive cell types may contribute. Nonetheless, we conclude that collateral targeting of IL-12 with anti-p40 mAbs in the treatment of psoriasis might carry more risk than benefit and even be counterproductive, which warrants further translational investigation.

## Methods

### Mice

C57BL/6 were purchased from Janvier (Saint Berthevin, France). *Il12rb2*^*−*/*−*^, *Il12a*^*−*/*−*^, *Ebi3*^*−/−*^, *Ifng*^*−/−*^ and *Il23a*^*−*/*−*^ were purchased from Jackson Laboratory (Bar Harbor, ME, USA) and Regeneron (Tarrytown, NY, USA), respectively. *Il12b*^*−/−*^ mice were purchased from Jackson Laboratory or provided by E. von Stebut and K. Schwonberg (Mainz, Germany). *Vd1*^*−/−*^ animals were provided by K. Kishihara (Nagasaki, Japan). Mice were kept in house under specific pathogen-free conditions. Animal experiments were approved by the Swiss Cantonal Veterinary Office (33/2010 and 68/2013).

### Aldara treatment

The 7- to 11-week-old female mice (cf. figure legends) of similar body weight and synchronized hair cycle were used for all experiments. Back skin of mice was shaved and depilated, and 48 h later 55 mg of Aldara cream (5% IMQ; 3M Pharmaceuticals, Maplewood, MN, USA) was applied daily for 2 or 5–6 constitutive days. When ear skin was used 7 mg of Aldara was applied on each ear for 6–7 constitutive days. Back skin or ear thickness was measured daily with a digital calliper. Skin inflammation is represented as a per cent change in the skin thickness compared with untreated skin on day 0. IL-12Fc/PBS treatment was performed by local subcutaneous injection of 200 ng of IL-12Fc (ref. 67) or PBS per each ear. Mice were injected every second day starting on day −1. Anti-IL-17A (17F3, BioXcell, West Lebanon, NH, USA) or isotype control (MOPC-21, BioXcell), anti-IL12p75 (R2-9A5, BioXcell) or isotype control (LTF-2, BioXcell) antibodies were injected intraperitoneally. Mice received 200 μg of antibodies every second day starting on day−1.

Group sizes were at least two experimental versus two control mice, but most of the time three versus three or more. Mouse numbers and experiment numbers are stated in the individual figure legends. All individual experiments showed the phenotypes depicted by the cumulative graphs. Mice of different experimental groups were mixed in the cages and the order in which individual mice were picked for Aldara treatment or any measurements was random. All experiments were blinded for disease induction and experimental read-outs.

### TEWL measurement

TEWL of dorsal skin was measured during the course of Aldara-induced plaque formation by use of an evaporimeter equipped with a closed chamber probe (Aquaflux AF200, Biox System Ltd, London, UK).

### Mouse skin leukocyte isolation

Back skin or ears were cut into small pieces and digested for 1 h at 37 °C in RPMI 1640 (PAN-Biotech, Aidenbach, Germany) medium containing 1 mg ml^−1^ collagenase type IV (Sigma-Aldrich, St Louis, MO, USA), 25 mM HEPES (Gibco, Thermo Fisher Scientific, Waltham MA, USA) and 0.1 mg ml^−1^ DNase (Sigma-Aldrich). Cells were filtered with a 70 μm cell strainer to receive single-cell suspension.

### Mouse keratinocyte isolation

For real-time quantitative PCR analysis, as well as NGS back skin was incubated for 2 h at 37 °C in HBSS buffer (Gibco) containing 2.4 mg ml^−1^ of dispase (Roche, Switzerland), next cut into small pieces and digested for another hour at 37 °C in HBSS buffer containing 10% FCS, 0.4 mg ml^−1^ collagenase type IV (Sigma-Aldrich) and 0.1 mg ml^−1^ DNase (Sigma-Aldrich). Cells were filtered with a 70 μm cell strainer to receive single-cell suspension.

### Bone marrow/neonatal thymocyte chimeras

Host animals received split dose (2 × 550 rad with 24 h interval) before receiving 5 × 10^6^ donor bone marrow together with 2 × 10^6^ donor neonatal thymocytes. Mice were kept for another 8 weeks to allow immune system reconstitution.

### Human skin biopsies and primary keratinocyte cell isolation

All donors signed written informed consent forms in accordance with the Code of Ethics of the World Medical Association (Declaration of Helsinki) for experiments involving humans (ethical approval number EK647). All samples were obtained from the University Hospital Zurich. Healthy human skin pieces (∼0.5–1 cm^2^) were incubated overnight at 4 °C in CnT07 (CELLnTEC) medium complemented with 1% penicillin (Gibco), 1% streptomycin (Gibco), 1% amphotericin B (Gibco) and 10 mg ml^−1^ dispase (Roche). Afterwards, the epidermis was separated manually and incubated in pre-warmed 0.25% trypsin-EDTA (Gibco) for ∼5 min. Keratinocytes were scratched off the epidermis and centrifuged at 1,500 r.p.m. for 5 min. The pellet was re-suspended in CnT07 medium complemented with antibiotics and antimycotic, and transferred to a culture flask. Medium was changed every 2 days until the cells reached 80–90% confluence and could be split for the first time.

### Human PBMC isolation

Blood samples from healthy volunteers were recruited via the blood donation centre, Zurich, Switzerland, with approval of the Cantonal Ethics Committee, Zurich. PBMCs were isolated by density gradient centrifugation using Lympholyte-H (Cedarlanes, Burlington, NC, USA). Cells were cultured for 3 days in RPMI 1640 (PAN-Biotech) with 10% FCS, 10 ng ml^−1^ IL-2 (PeproTech, Rocky Hill, NJ, USA), 10 ng ml^−1^ IL-12 (PeproTech), 1 μg ml^−1^ anti-human CD28 antibody (CD28.2, BD Pharmingen, San Diego, CA, USA) and 1 μg ml^−1^ plate-bound anti human CD3 antibody (OKT3, BioXcell). Next, cells were washed and lysed for protein extraction. Human monocytes were enriched using magnetically labelled anti-CD14 MicroBeads in combination with the AutoMACS system (both Miltenyi Biotec, Auburn, CA, USA). CD14-positive cells were collected, washed and lysed for protein extraction.

### Cell lines

17D1 hybridoma (anti-mouse Vγ5/Vδ1, Vγ6/Vδ1 and Vγ1/Vδ1; rat immunoglobulin IgM) was kindly provided by Robert R Tigelaar. Cells were cultured in RPMI 1640 medium supplemented with 10% fetal bovine serum (Biochrome AG, Berlin, Germany), 10 mM HEPES, penicillin (Gibco), streptomycin (Gibco), glutamine (Gibco), sodium pyruvate (Gibco) and non-essential amino acids (Gibco). After 3 and 6 days of culture supernatant was collected, centrifuged, filtered and used for staining.

### Flow cytometry

For surface and intracellular staining following antibodies coupled to the appropriate fluorochromes were titrated and used in saturating concentration: rat anti-mouse CD45 (30-F11, BD, Franklin Lakes, NJ, USA, 1:800); rat anti-mouse CD3 (17A2, eBioscience, San Diego, CA, USA, 1:100); rat anti-mouse CD11b (M1/70, BioLegend, San Diego, CA USA, 1:400); rat anti-mouse Ly6G (1A8, BD, 1:400); armenian hamster anti-mouse γδ TCR (GL3, eBioscience, 1:400); armenian hamster anti-mouse Vγ4 TCR (UC3-10A6, BioLegend, 1:400); syrian hamster anti-mouse Vγ5 TCR (536, BioLegend, 1:400); rat anti-mouse IL-17A (TC11-18H10, BioLegend, 1:200); rat anti-mouse/-human CD49f (GoH3, BioLegend, 1:300); and rat anti-mouse CD34 (RAM34, eBioscience, 1:100). Cells were incubated for 10 min at 4 °C with 1 μg of rat anti-mouse CD16/CD32 (93, eBioscience) antibodies, followed by 25 min surface staining at 4 °C. For intracellular staining, mouse cells were stimulated with 50 ng ml^−1^ phorbol 12-myristate 13-acetate (Applichem, Darmstadt, Germany) and 500 ng ml^−1^ ionomycin (Invitrogen, Thermo Fisher Scientific, Carlsbad, CA USA) in the presence of GolgiPlug (BD Biosciences) for 2 h. After surface staining cells were fixed and permeabilized according to the manufacturer's (BD Biosciences) recommendations and next stained intracellularly. For 17D1 staining of Vγ5/Vδ1^+^ and Vγ6/Vδ1^+^ cells, cells were first preincubated with rat anti-mouse γδ TCR antibody (GL3, eBioscience, 1:400) for 20 min in 4 °C, next washed and incubated with 100 μl of 17D1 hybridoma supernatant for the next 20 min followed by washing and 20 min incubation with fluorochrome-conjugated secondary antibody goat anti-rat IgM (Jackson ImmunoResearch Laboratories, West Grove, PA, USA, 1:200). Samples were analysed with a BD FACS LSR II Fortessa. Post-acquisition analysis was done with FlowJo (Tree Star, Ashland, OR, USA) software.

### Cell sorting

γδT cells were isolated from the back skin of Aldara-treated WT or *Il12rb2*^*−/−*^ mice on day 6 post treatment. The whole-cell suspension was stained with the following: rat anti-mouse CD45 (30-F11, BD, 1:800); rat anti-mouse CD11b (M1/70, BioLegend, 1:400); rat anti-mouse CD3 (17A2, eBioscience, 1:100); rat anti-mouse γδ TCR (GL3, eBioscience, 1:400); rat anti-mouse Vγ5 (536, BioLegend, 1:400); rat anti-mouse Vγ4 (UC3-10A6, BioLegend, 1:400) or 17D1 antibodies. Cells were sorted with BD FACS Aria III using 70 μm nozzle. Murine keratinocytes were isolated from naive skin or treated with Aldara for 2 days. The whole-cell suspension was stained with rat anti-mouse CD45 (30-F11, BD, 1:800), rat anti-mouse CD34 (RAM34, eBioscience, 1:100) and rat anti-mouse/-human CD49f (GoH3, BioLegend, 1:300) antibodies. Cells were sorted with BD FACS Aria III using 100 μm nozzle.

### RNA extraction and real-time quantitative PCR

Total RNA was isolated from the back skin of Aldara-treated animals, mouse skin-sorted leukocytes or keratinocytes, with a Pure Link RNA Micro Kit (Invitrogen) or RNeasy Plus Micro Kit (QIAGEN, Valencia, CA, USA). cDNA was prepared using SuperScript III reverse transcriptase (Invitrogen). Mouse gene expression was measured by real-time quantitative PCR analysis using the CFX 384 Real-Time detection system (Bio-Rad, Hercules, CA, USA) with SYBR Green Supermix (Bio-Rad). Sequences for PCR primers can be found in Supplementary Table 1. Transcript expression was normalized to the *Polr2a* or *Gapdh* house-keeping gene and represented as either 2^*−*ΔC^_T,_ (ΔC_T_=C_T_ gene of interest−C_T_ house-keeping gene) in the case of *Il12rb2* expression or 2^*−*ΔΔC^_T_ (ΔΔC_T_=ΔC_T_−ΔC_Control_), for all other transcripts.

### Histochemistry

Skin tissue samples were fixed in 4% paraformaldehyde and embedded in paraffin. Mouse skin sections were stained with haematoxylin and eosin according to standard protocols. Deparaffinized human skin sections were submitted to antigen retrieval with citrate buffer pH 6 (DAKO, Hamburg, Germany) followed by blocking and staining with polyclonal rabbit anti-human IL-12Rβ2 antibody (Novus Biologicals, Littleton, CO, USA; 0.7 μg ml^−1^) or total rabbit IgG (Sigma, 0.7 μg ml^−1^) using Dako Envision+Dual Link System-HRP (DAB+) staining (DAKO) and following the manufacturer's procedure. We recorded digital images of tissue sections using an Olympus BX41 light microscope with an Olympus ColorView IIIu camera and Olympus Cell B image acquisition software. For immunofluorescence staining of human skin sections we co-stained with monoclonal mouse anti-human cytokeratin 14 (K14) antibody (LL002, Abcam, Cambridge, UK, 1:300). Secondary antibodies were Alexa Fluor 546-conjugated goat anti-rabbit IgG or Alexa Fluor 633-conjugated goat anti-mouse IgG (Invitrogen, 1:500). Specimens were mounted in 4,6-diamidino-2-phenylindole-containing mounting medium (Invitrogen) and analysed with a SP5 Leica confocal laser scanning microscope (SP5; Leica, Wetzlar, Germany) using an argon and a helium laser with a × 20 objective (oil immersion, numerical aperture 0.7, Leica) and Imaris imaging software (version 7.5.1; Bitplane, Zurich, Switzerland).

### Western blot analysis of human IL-12Rβ2

Human primary keratinocyte monolayers, monocytes and PBMCs were lysed with the cell lysis buffer (Cell Signalling, Danvers, MA USA), complemented with complete EDTA-free protease inhibitor cocktail (Roche) and phosphatase inhibitor cocktail (Roche). Lysates were incubated for 15 min on ice followed by centrifugation at 4 °C for 15 min at maximum speed. Supernatants were collected and total protein content was measured using bicinchoninic acid protein assay (Thermo Scientific, Waltham, MA USA). Equal amounts of cell lysates were separated by 10% SDS–PAGE and transferred to nitrocellulose (Bio-Rad) by wet blotting. Next, membranes were stained with polyclonal rabbit anti-human IL-12Rβ2 antibodies (Novus Biologicals, 1:1,000) or monoclonal rabbit anti-mouse/human β-actin antibodies (13E5, Cell Signalling, 1:2,000) followed by staining with peroxidase-conjugated mouse antibodies against rabbit IgG (Jackson ImmunoResearch, 1:50,000). The signal was visualized using SuperSignal West Pico chemiluminescent substrate (Thermo Scientific).

### NGS of human samples

Two independent NGS analysis were performed for human primary keratinocytes. For the first sequencing analysis, cells were isolated from two healthy donors and stimulated for 16 h with 25 ng ml^−1^ of TNF (PeproTech) followed by 6 h stimulation with 100 ng ml^−1^ IL-12 (PeproTech). For the second sequencing analysis cells were isolated from one healthy donor and stimulated in triplicates for 16 h with 25 ng ml^−1^ of TNF (PeproTech) followed by 12 h stimulation with 100 ng ml^−1^ IL-12 (PeproTech). Total RNA was isolated with RNeasy Plus Micro Kit (QIAGEN) according to the manufacturer's instructions. NGS was performed by the Functional Genomics Center in Zurich or by the Quantitative Genomics Facility in Basel. Reads were quality-checked with FastQC. Low-quality ends were clipped (3 bases from the start and 10 bases from the end). Trimmed reads were aligned to the reference genome and transcriptome (FASTA and GTF files, respectively, downloaded from the Ensembl GRCm38) with STAR version 2.3.0e_r291 (ref. 68) with default settings. Distribution of the reads across genomic isoform expression was quantified using the R package GenomicRanges^69^ from Bioconductor Version 3.0. Differentially expressed genes were identified using the R package edgeR (ref. 70) from Bioconductor Version 3.0. Only transcripts with a read count above 10 in at least 50% of at least one of the two groups were retained in the two-group comparisons. Pathway analysis was performed with Metacore from Thomson Reuters (New York, NY, USA).

### NGS of mouse samples

WT and *Il12rb2*^*−/−*^ keratinocytes were isolated from naive animals or treated for 2 days with Aldara. Cells were sorted and total RNA was isolated with RNeasy Plus Micro Kit (QIAGEN) according to the manufacturer's instructions. NGS was performed by the Quantitative Genomics Facility in Basel.

### Statistics

Differences between ≥3 groups were evaluated with one-way or two-way analysis of variance (ANOVA) with Bonferroni's post test. Differences between two sets of data were evaluated using unpaired two-tailed *t*-test. Correlation calculation between two parameters has been performed using Pearson's *r* (comparable distribution was confirmed via quantile–quantile plots); *P<0.05, **P<0.01, ***P<0.001, NS (not significant). Data were analysed using Prism software (GraphPad Software, Inc.).

### Data availability

Sequence data that support the findinfgs of this study have been deposited in the European Nucleotide Archive with the primary accession code PRJEB15422. The authors declare that all other data supporting the findings of this study are available within the article and its Supplementary Information files.

## Additional information

**How to cite this article:** Kulig, P. *et al*. IL-12 protects from psoriasiform skin inflammation. *Nat. Commun.* **7,** 13466 doi: 10.1038/ncomms13466 (2016).

**Publisher's note**: Springer Nature remains neutral with regard to jurisdictional claims in published maps and institutional affiliations.

## Supplementary Material

Supplementary Information Supplementary Figures 1-14 and Supplementary Table 1.
