# Supplementary material for: IL-12 protects from psoriasiform skin inflammation
Source: Nat Commun. 2016 Nov 28;7:13466. doi: 10.1038/ncomms13466 (PMC5133729; doi:10.1038/ncomms13466)
Supplement: Supplementary Information — Supplementary Figures 1-14 and Supplementary Table 1. [file ncomms13466-s1.pdf]

Supplementary Fig. 1

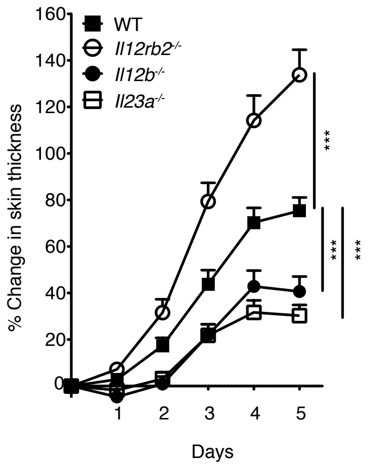

Supplementary Figure 1. Psoriatic plaque formation in mice deficient in IL-12/23p40, IL-23 and IL-12R. WT, *Il12b*$^{-/-}$, *Il23a*$^{-/-}$ and *Il12rb2*$^{-/-}$ mice were treated with Aldara for 6 days. Back skin inflammation during the whole course of the disease represented as a percent change in skin thickness compared to untreated skin on day 0. Cumulative graph of 4 independent experiments (n=21 per WT, n=15 per *Il12rb2*$^{-/-}$, n=16 per *Il12b*$^{-/-}$, n=9 per *Il23a*$^{-/-}$, average mean ± s.e.m.). Each data point represents individual mouse. *p<0.05, **p<0.01, ***p<0.001 (Two Way ANOVA with Bonferroni post test).

Supplementary Fig. 2

a

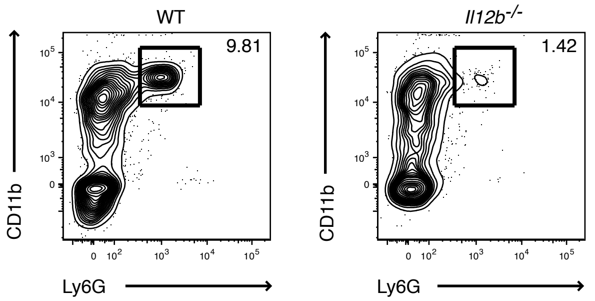

b

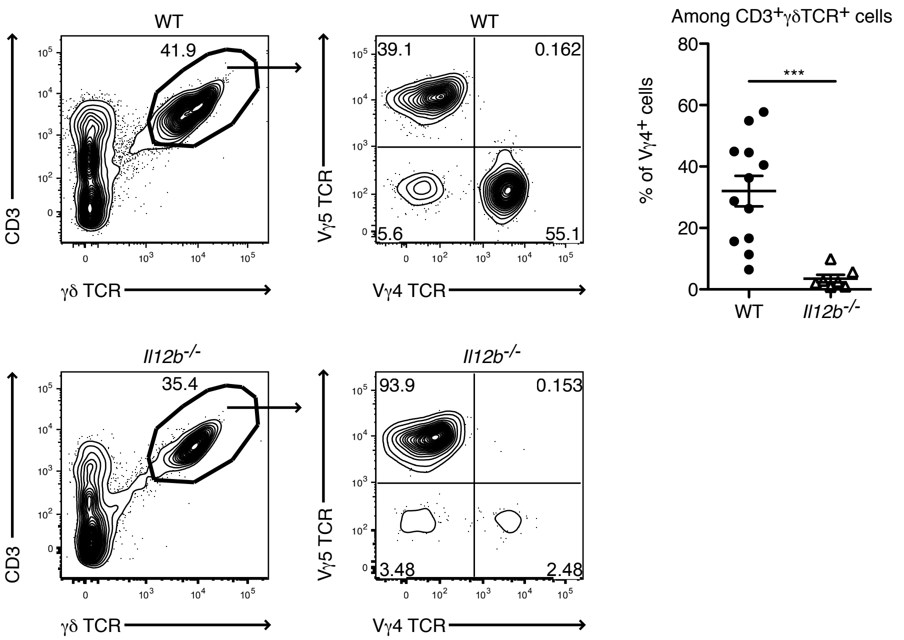

Supplementary Figure 2. Analysis of inflamed skin in IL-12/23p40 deficient mice. (**a,b**) WT and *Il12b*-/- mice were treated with Aldara for 6 days. Flow cytometry analysis of inflamed skin; cells were gated on CD45+ leukocytes and analyzed for the presence of (**a**) neutrophils and (**b**) skin infiltrating Vγ4+ γδT cells. (**b**) Cumulative graph of 3 independent experiments, (n=12 per WT, n=7 per *Il12b*-/-, average mean ± s.e.m.). Each data point represents individual mouse. *p<0.05, **p<0.01, ***p<0.001 ((**b**)unpaired two tailed t-test).

Supplementary Fig. 3

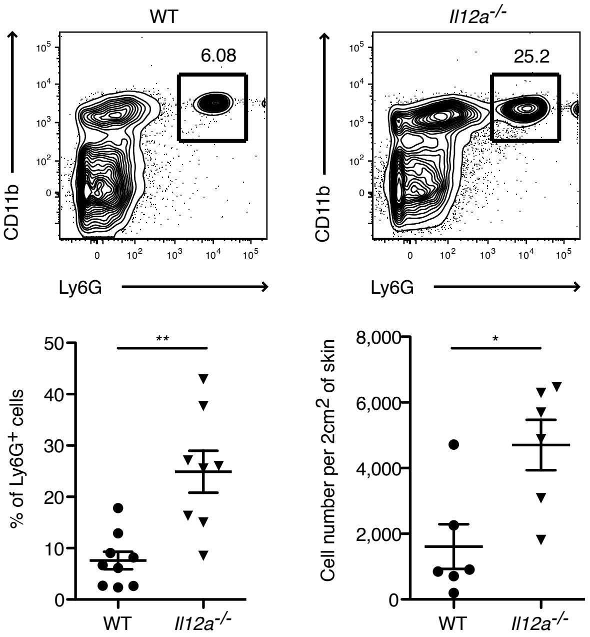

Supplementary Figure 3. Analysis of Aldara treated skin in WT and *Il12a*$^{-/-}$ mice. Mice were treated with Aldara for 6 constitutive days. Flow cytometry analysis of inflamed back skin; cells were gated on $CD45^{+}$ leukocytes and analyzed for the presence of neutrophils. Cumulative graph of 2-3 independent experiments, (n=6-9 per WT, n=6-8 per *Il12a*$^{-/-}$, average mean ± s.e.m.). Each data point represents individual mouse. *p<0.05, **p<0.01**, ***p<0.001 (unpaired two tailed t-test).

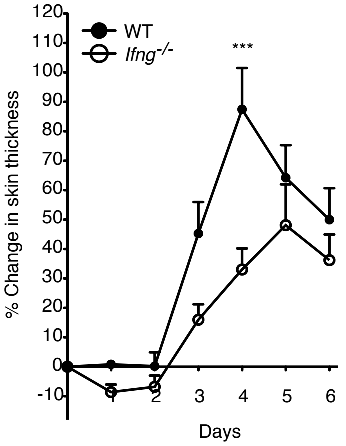

Supplementary Figure 4. Impact of IFN-γ deficiency on psoriatic plaque formation. WT and *Ifng*$^{-/-}$ mice were treated with Aldara for 6 days. Back skin inflammation during the whole course of the disease represented as a percent change in skin thickness compared to untreated skin on day 0. Cumulative graph of 2 independent experiments, (n=6 per WT, n=7 per *Ifng*$^{-/-}$, average mean ± s.e.m.). Each data point represents individual mouse. *p<0.05, **p<0.01, ***p<0.001 (Two Way ANOVA with Bonferroni post test).

Supplementary Fig. 5

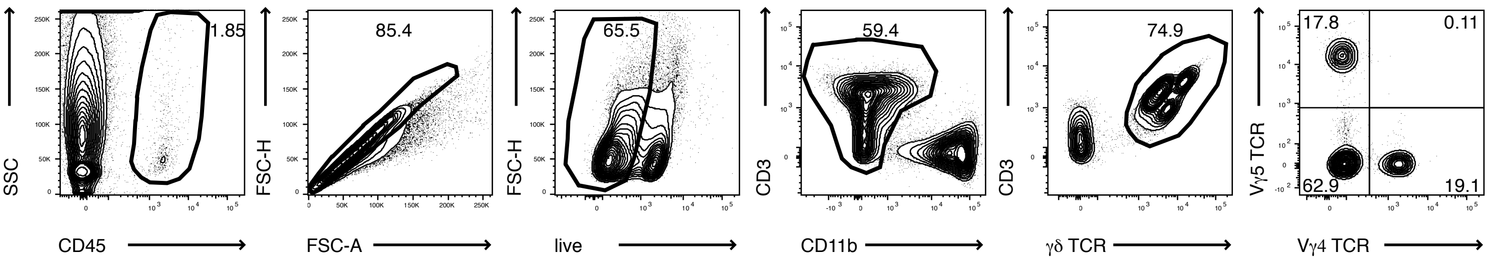

Supplementary Figure 5. Representative flow cytometry gating strategy for the mouse skin. Cells were gated on CD45$^+$/singlets$^+$/live$^+$/CD11b$^-$ leukocytes and analyzed for the presence of skin resident and skin infiltrating γδT cells.

Supplementary Fig. 6

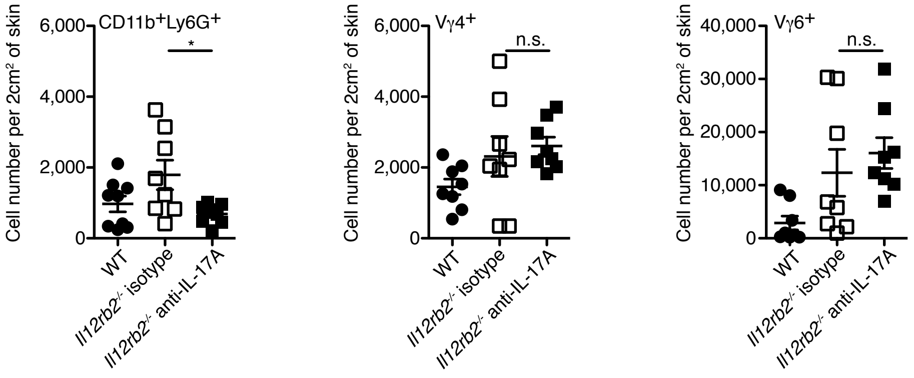

Supplementary Figure 6. Analysis of inflamed skin in animals treated with anti-IL-17A antibody. WT and *Il12rb2*$^{-/-}$ mice were treated with Aldara, 200 ug of anti-IL-17A antibody or isotype control was injected i.p. into *Il12rb2*$^{-/-}$ mice every second day starting on day -1. Flow cytometry analysis of inflamed skin; cells were gated on CD45^{+} leukocytes and analyzed for the presence of neutrophils and skin infiltrating Vγ4^{+} and Vγ6^{+} γδT cells. Cumulative graph of 2 independent experiments, (n=8 per WT, n=8 per *Il12rb2*$^{-/-}$, average mean ± s.e.m.). Each data point represents individual mouse.*p<0.05, **p<0.01, ***p<0.001 (unpaired two tailed t-test).

Supplementary Fig. 7

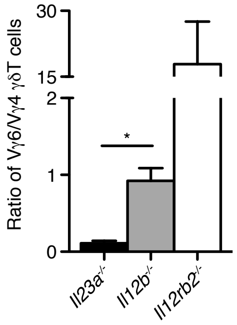

Supplementary Figure 7. Effector γδT cell distribution in Aldara treated skin of IL-23, IL-12/23p40 and IL-12R deficient animals. *Il23a*$^{-/-}$, *Il12b*$^{-/-}$ and *Il12rb2*$^{-/-}$ mice were treated with Aldara for 6 days followed by flow cytometry analysis of Aldara treated skin. The graph depicts the ratio of Vγ6/Vγ4 γδT cells. Cumulative graph of 3 experiments, (n=4 per *Il12rb2*$^{-/-}$, n=9 per *Il12b*$^{-/-}$, n=3 per *Il23a*$^{-/-}$, average mean ± s.e.m.). *$p<0.05$, **$p<0.01$, ***$p<0.001$ (unpaired two tailed t-test).

Supplementary Fig. 8

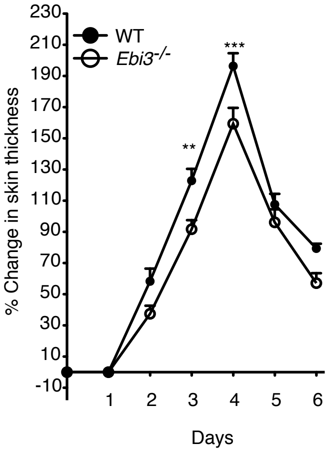

Supplementary Figure 8. Psoriatic plaque formation in Aldara treated *Ebi3*$^{-/-}$ animals. WT and *Ebi3*$^{-/-}$ mice were treated with Aldara for 6 days. The graph shows back skin swelling during the whole course of the disease represented as a percent change in skin thickness compared to untreated skin on day 0. Cumulative graph of 2 independent experiments, (n=8 per WT and *Ebi3*$^{-/-}$, average mean ± s.e.m.). Each data point represents individual mouse. *p<0.05, **p<0.01, ***p<0.001 (Two Way ANOVA with Bonferroni post test).

Supplementary Fig. 9

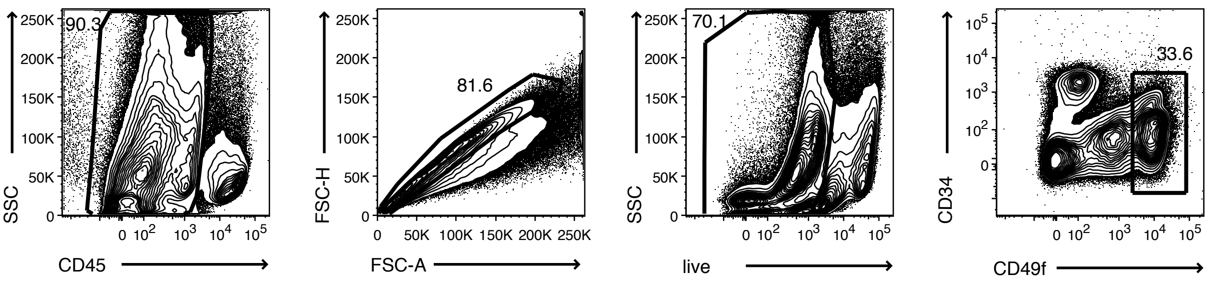

Supplementary Figure 9. Mouse keratinocyte gating strategy for cell sorting. Representative dot plots displaying staining of murine skin for keratinocyte sort isolation. Cells negative for CD45 and CD34 marker and positive for CD49f were sorted for further analysis.

Supplementary Fig. 10

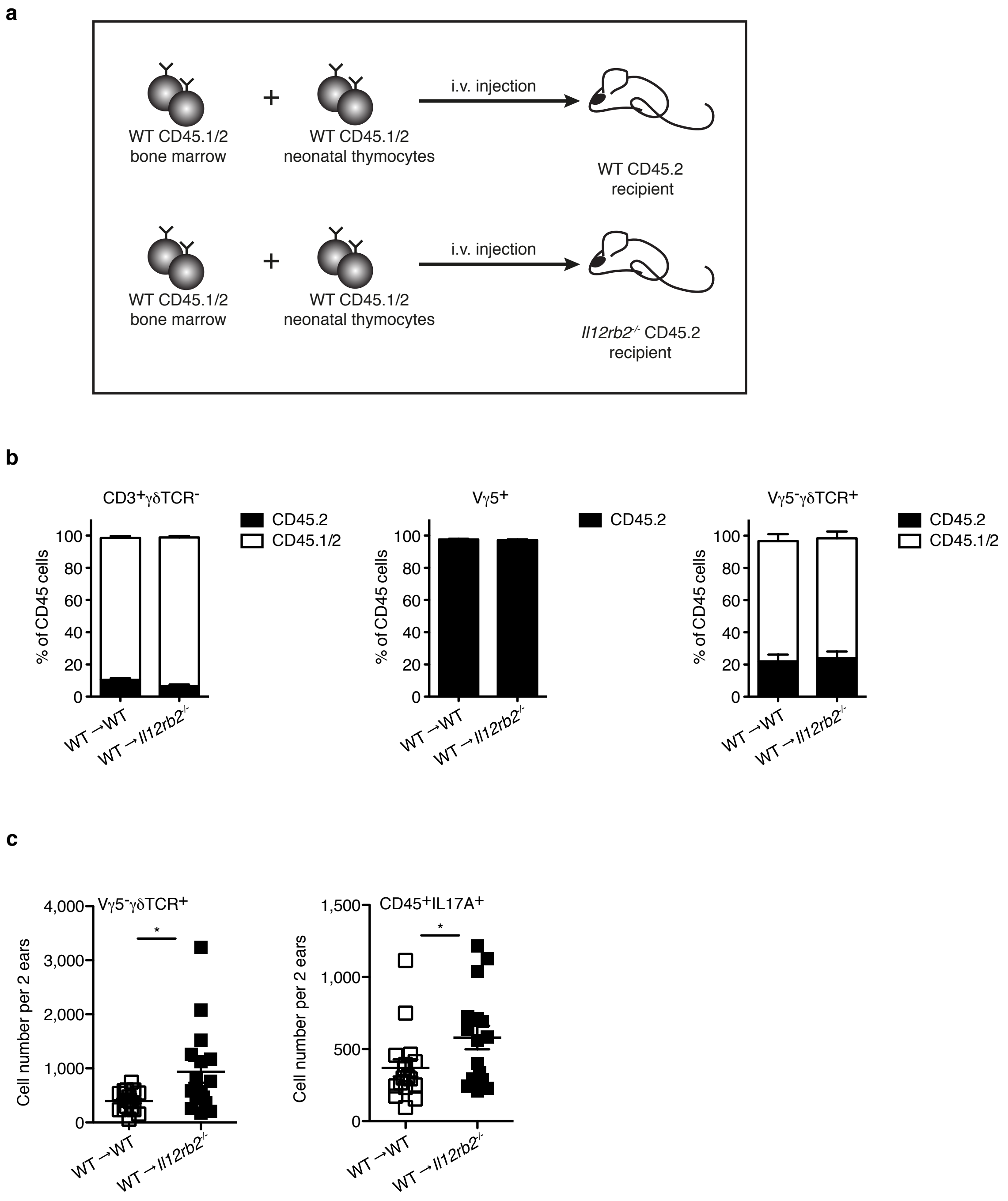

Supplementary Figure 10. Analysis of inflamed skin in bone marrow/thymocytes chimeras. (**a**) Representative scheme of reconstituted animals with donor bone marrow and neonatal thymocytes. (**b,c**) Mice were treated with Aldara for 7 days. On day 8 ear inflitrated cells were isolated and analyzed, (**b**)T cell reconstitution in Aldara treated skin of chimeric animals, (**c**) flow cytometry analysis of inflamed ears; absolute numbers of skin infiltrating leukocytes are depicted. (**b, c**) Cumulative graph of 4 independent experiments, (n=17 per WT into WT, n=16 WT into *Il12rb2*$^{-/-}$, average mean ± s.e.m.). Each data point represents individual mouse. *p<0.05, **p<0.01, ***p<0.001 ((**c**) unpaired two tailed t-test).

Supplementary Fig. 11

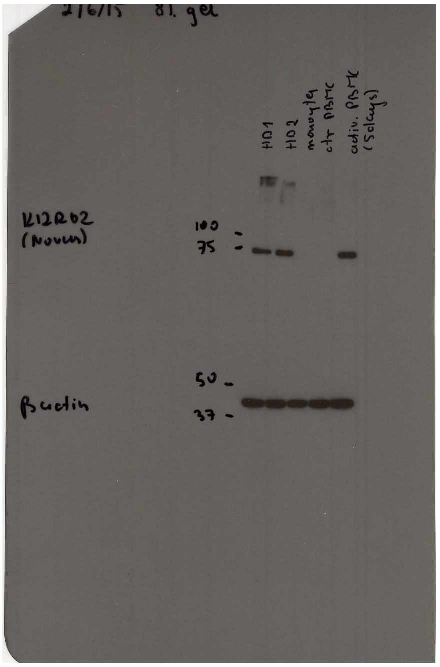

Supplementary Figure 11. IL-12Rβ2 expression in human cells. Immunoblot analysis of IL-12Rβ2 in human primary keratinocytes. Human monocytes, naïve and activated PBMC were used as negative and positive controls. Figure shows uncropped western blot data.

Supplementary Fig. 12

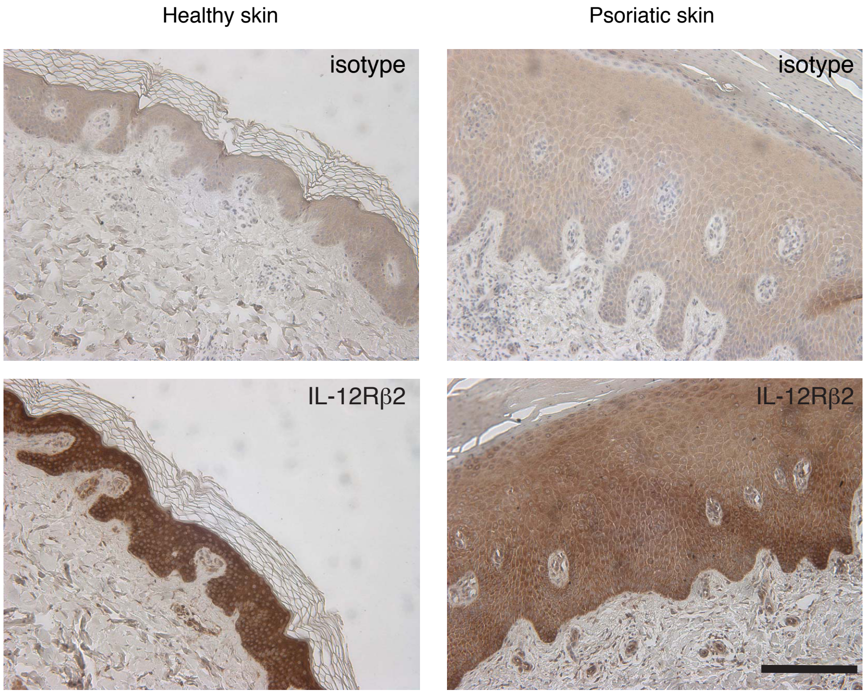

Supplementary Figure 12. IL-12Rβ2 expression in human skin. Skin sections from healthy human donor and psoriatic patient were stained with antibodies against human IL-12Rβ2 or isotype control for immunohistochemistry, scale bar: 200 μm.

Supplementary Fig. 13

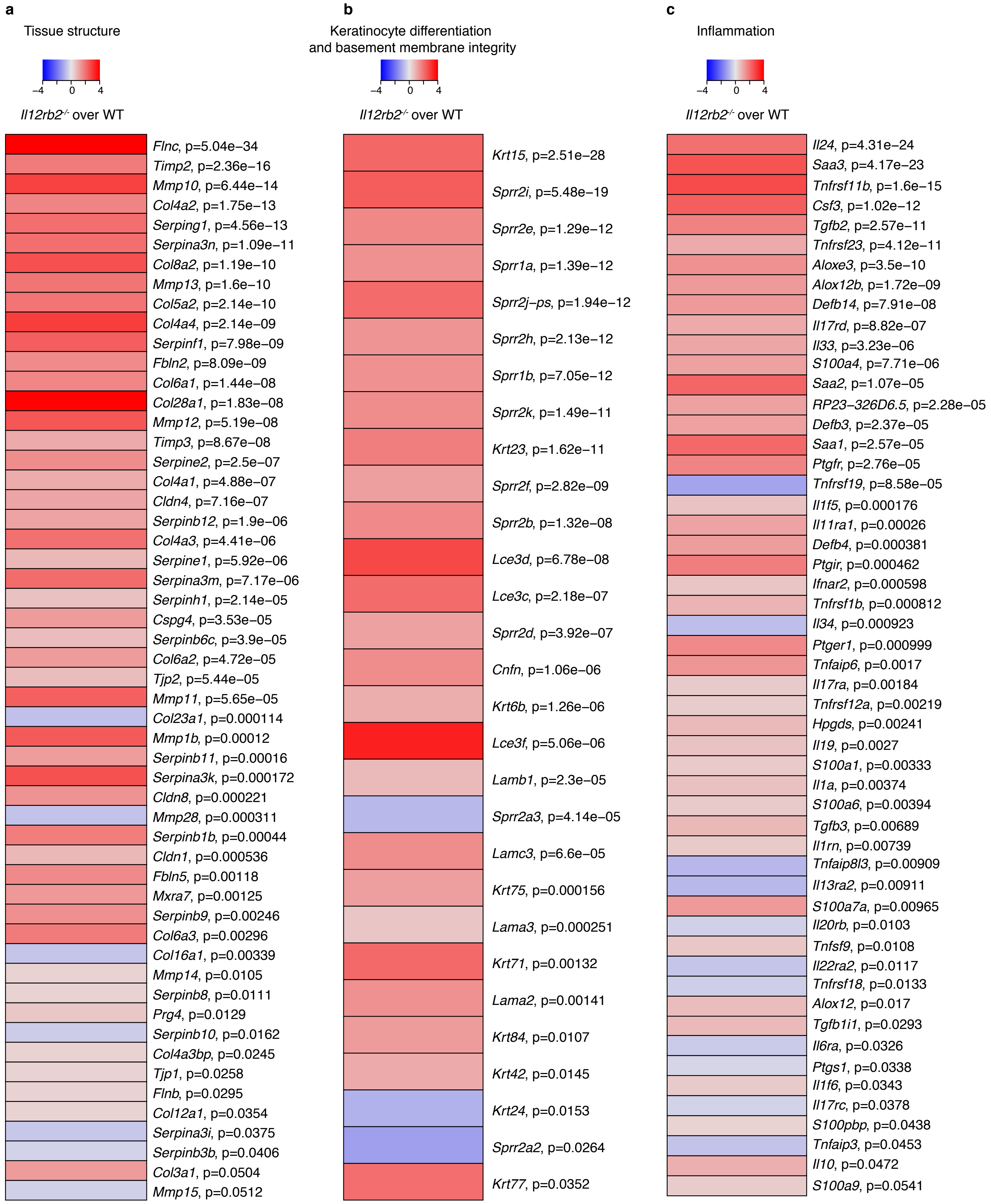

Supplementary Figure 13. Next generation sequencing of mouse keratinocytes isolated from Aldara treated animals. Heat maps showing list of genes affected by disrupted IL-12 signalling. Significance is shown alongside, (unpaired two tailed t-test).

Supplementary Fig. 14

Pathway Maps

Cluster	Term	Count	p-value
1	Cytoskeleton remodeling keratin filaments	5	1.411E-04
	Immune response MIF mediated glucocorticoid regulation	4	2.780E-04
	Development regulation of epithelial to mesenchymal transition (EMT)	6	2.781E-04
	Development TGF-b dependent induction of EMT via MAPK	5	5.067E-04
	Oxidative phosphorylation	7	7.086E-04
	Immune response ETV3 affect on SCF1 promoted macrophage differentiation	4	1.151E-03
	Development MAG-dependent inhibition of neurite outgrowth	4	1.778E-03
	Role of red blood cell adhesion to endothelium in vaso-occlusion in Sickle cell disease	4	1.778E-03
	Apoptosis and survival Apoptotic TNF-family pathways	4	2.854E-03
	Development TGF-beta-dependent induction of EMT via RhoA, PI3K and ILK.	4	3.983E-03
2	Cell adhesion ECM remodeling	10	8.207E-10
	Cell adhesion Chemokines and adhesion	9	4.920E-06
	Cell adhesion Cell-matrix glycoconjugates	6	7.699E-06
	Cytoskeleton remodeling TGF, WNT and cytoskeletal remodeling	9	1.162E-05
	Expression targets of Tissue factor signaling in cancer	4	1.567E-04
	Development Regulation of epithelial-to-mesenchymal transition (EMT)	6	1.594E-04
	Cytoskeleton remodeling Cytoskeleton remodeling	7	3.211E-04
	Cell adhesion Plasmin signaling	4	9.864E-04
	Signal transduction Cyclic AMP signaling	4	1.350E-03
	Development PDGF signaling via MAPK cascades	4	2.986E-03
3	dCTP/dUTP metabolism	13	2.922E-08
	Cell cycle Start of DNA replication in early S phase	9	4.953E-08
	Cell cycle Role of APC in cell cycle regulation	7	1.009E-05
	dATP/dITP metabolism	11	2.045E-05
	Cell cycle The metaphase checkpoint	7	2.300E-05
	dGTP metabolism	9	2.779E-05
	TTP metabolism	9	3.150E-05
	Cell cycle Transition and termination of DNA replication	6	5.043E-05
	Immune response Classical complement pathway	7	2.975E-04
	Apoptosis and survival DNA-damage-induced apoptosis	4	3.974E-04
4	Immune response IL-6-induced acute-phase response in hepatocytes	2	1.638E-03
	Development BMP7 in brown adipocyte differentiation	2	1.921E-03
	Immune response IL-13 signaling via JAK-STAT	2	2.441E-03
	Role of IL-23/ T17 pathogenic axis in psoriasis	2	3.656E-03
	Immune response IL-17 signaling pathways	2	4.495E-03
	Immune response Oncostatin M signaling via JAK-Stat in mouse cells	1	3.008E-02
	Immune response Oncostatin M signaling via JAK-Stat in human cells	1	3.337E-02
	Development Thrombopoetin signaling via JAK-STAT pathway	1	3.665E-02
	Expression targets of Tissue factor signaling in cancer	1	3.665E-02
	Cell adhesion Gap junctions	1	4.966E-02
6	Breast cancer (general schema)	8	5.929E-06
	Apoptosis and survival Anti-apoptotic TNFs/NF-kB/Bcl-2 pathway	8	7.169E-06
	Upregulation of MITF in melanoma	7	2.365E-05
	Cell adhesion Gap junctions	6	7.811E-05
	Colorectal cancer (general schema)	6	7.811E-05
	Development Regulation of epithelial-to-mesenchymal transition (EMT)	8	1.682E-04
	Immune response CD40 signaling	8	1.878E-04
	Immune response Th17 cell differentiation	6	1.925E-04
	Development TGF-beta-dependent induction of EMT via SMADs	6	1.925E-04
	Cell adhesion Tight junctions	6	2.263E-04

GO Processes

Cluster	Term	Count	p-value
1	Selenocysteine metabolic process	19	2.630E-15
	Viral transcription	20	2.280E-14
	SRP-dependent cotranslational protein targeting to membrane	19	1.680E-13
	Viral gene expression	20	1.697E-13
	Nuclear-transcribed mRNA catabolic process, nonsense-mediated decay	20	2.263E-13
	Cotranslational protein targeting to membrane	19	2.302E-13
	Selenium compound metabolic process	19	2.688E-13
	Serine family amino acid metabolic process	20	5.967E-13
	Multi-organism metabolic process	20	6.822E-13
	Protein targeting to ER	19	8.823E-13
2	Anatomical structure morphogenesis	107	9.639E-24
	System development	146	4.547E-22
	Single-organism developmental process	168	9.011E-22
	Developmental process	168	3.868E-21
	Single-multicellular organism process	169	4.726E-21
	Multicellular organismal development	155	6.348E-21
	Cell differentiation	128	6.398E-21
	Anatomical structure development	156	1.059E-20
	Cellular developmental process	129	9.838E-20
	Regulation of developmental process	97	1.149E-19
3	Single-organism metabolic process	245	1.436E-21
	DNA replication	40	1.574E-20
	Single-organism cellular process	439	1.381E-18
	DNA metabolic process	73	1.481E-18
	Response to stress	203	1.913E-18
	Mitotic cell cycle	74	3.557E-18
	Mitotic cell cycle process	68	1.519E-16
	Small molecule metabolic process	141	2.328E-16
	Cell cycle process	84	7.637E-16
	Cell cycle	94	1.507E-15
4	Keratinization	10	4.451E-17
	Keratinocyte differentiation	11	1.298E-15
	Peptide cross-linking	9	1.438E-14
	Epidermal cell differentiation	11	9.880E-14
	Skin development	11	7.434E-12
	Epidermis development	11	5.417E-11
	Epithelial cell differentiation	13	1.354E-10
	Epithelium development	15	3.920E-09
	Tissue development	17	3.408E-08
	Response to lipid	13	6.321E-07
6	Single-multicellular organism process	307	3.035E-22
	System development	254	1.083E-21
	Cellular response to chemical stimulus	187	7.619E-21
	Anatomical structure development	277	1.170E-20
	Regulation of cellular component movement	83	6.099E-20
	Regulation of cell motility	78	4.185E-19
	Multicellular organismal development	269	6.179E-19
	Positive regulation of biological process	283	9.737E-19
	Regulation of locomotion	81	1.385E-18
	Regulation of cell migration	74	3.198E-18

Process Networks

Cluster	Term	Count	p-value
1	Translation Translation initiation	21	5.213E-10
	Translation Elongation-Termination	20	6.129E-07
	Cell adhesion Amyloid proteins	13	7.697E-04
	Inflammation IL-6 signaling	9	2.125E-03
	Cell adhesion Platelet-endothelium-leucocyte interactions	11	2.961E-03
	Cell adhesion Cell-matrix interactions	12	4.600E-03
	Development Skeletal muscle development	9	7.519E-03
	Development EMT_Regulation of epithelial-to-mesenchymal transition	12	7.605E-03
	Signal transduction NOTCH signaling	12	1.091E-02
	Cytoskeleton Intermediate filaments	6	1.275E-02
2	Cell adhesion Cell-matrix interactions	17	9.844E-07
	Cell adhesion Platelet-endothelium-leucocyte interactions	15	1.916E-06
	Proteolysis ECM remodeling	9	4.805E-05
	Development Blood vessel morphogenesis	15	5.051E-05
	Development Ossification and bone remodeling	12	7.102E-05
	Proteolysis Connective tissue degradation	10	1.326E-04
	Signal transduction WNT signaling	12	2.229E-04
	Cell adhesion Integrin-mediated cell-matrix adhesion	13	3.593E-04
	Development Hedgehog signaling	13	1.774E-03
	Development Neurogenesis Axonal guidance	12	2.267E-03
3	Cell cycle S phase	26	9.457E-13
	Cell cycle Core	21	6.740E-11
	DNA damage BER-NER repair	14	9.200E-06
	Cytoskeleton Spindle microtubules	13	4.304E-05
	DNA damage DBS repair	13	8.295E-05
	DNA damage Checkpoint	12	6.137E-04
	Cell cycle Mitosis	15	6.245E-04
	DNA damage Core	5	2.093E-03
	DNA damage MMR repair	7	2.689E-03
	Cell cycle G1-S	12	6.212E-03
4	Immune response Th17-derived cytokines	4	6.922E-05
	Proteolysis ECM remodeling	2	1.696E-02
	Inflammation IL-13 signaling pathway	2	1.929E-02
	Inflammation Amphoterin signaling	2	3.132E-02
	Proteolysis Connective tissue degradation	2	3.181E-02
	Inflammation IL-6 signaling	2	3.181E-02
	Chemotaxis	2	4.117E-02
	Cell adhesion Cell junctions	2	5.567E-02
	Muscle contraction	2	6.255E-02
	Cell adhesion Leucocyte chemotaxis	2	8.341E-02
6	Development EMT_Regulation of epithelial-to-mesenchymal transition	22	1.353E-05
	Proliferation Positive regulation cell proliferation	21	3.293E-05
	Cell adhesion Cell junctions	17	5.472E-05
	Cell adhesion Integrin-mediated cell-matrix adhesion	17	1.482E-03
	Cell adhesion Platelet-endothelium-leucocyte interactions	14	3.397E-03
	Development Ossification and bone remodeling	13	3.676E-03
	Cell cycle G1-S Growth factor regulation	15	3.780E-03
	Cytoskeleton Regulation of cytoskeleton rearrangement	14	5.331E-03
	Cytoskeleton Intermediate filaments	8	7.658E-03
	Proteolysis Connective tissue degradation	10	9.359E-03

Supplementary Figure 14. Next generation sequencing of mouse keratinocytes isolated from Aldara treated WT and *Il12rb2$^{-/-}$* animals. The enriched pathway maps, gene ontology and process networks cathegories for differentially expressed genes are shown. Metacore analysis of cluster 5 was excluded: combined genes were too functionally heterogeneous to produce relevant results.

Supplementary Table 1

PRIMERS	SEQUENCE
mouse Il12rb2	5'-TGT GGG GTG GAG ATC TCA GT
	5'-TCT CCT TCC TGG ACA CAT GA
mouse Vg6	5'-GAT CCA AGA GGA AAG GAA AGA CGG C
	5'-AAG GAG ACA AAG GTA GGT CCC AGC
mouse Cxcl9	5'-ATT TCA TCA CGC CCT TGA GCC T
	5'-AGC CAG ACA GCT GTT GTG CAT T
mouse Ccl20	5'-AAC TGGGTG AAA AGG GCT GT
	5'-GTC CAA TTC CAT CCC AAA AA
mouse Tnfa	5'-CTG TAG CCC ACG TCG TAG C
	5'-TTG AGA TCC ATG CCG TTG
mouse Il17a	5'-ATC AGG ACG CGC AAA CAT GA
	5'-TTG GAC ACG CTG AGC TTT GA
mouse Il17f	5'-TGC TAC TGT TGA TGT TGG GAC
	5'-AAT GCC CTG GTT TTG GTT GAA
mouse Il22	5'-ATG AGT TTT TCC CTT ATG GGG AC
	5'-GCT GGA AGT TGG ACA CCT CAA
mouse Il1b	5'-GAA ATG CCA CCT TTT GAC AGT G
	5'-TGG ATG CTC TCA TCA GGA CAG
mouse Ifng	5'-GCA TTC ATG AGT ATT GCC AAG
	5'-GGT GGA CCA CTC GGA TGA
mouse Defb1	5'-AGG TGT TGG CAT TCT CAC AAG
	5'-GCT TAT CTG GTT TAC AGG TTC CC
mouse Defb2	5'-TAT GCT GCC TCC TTT TCT CA
	5'-GAC TTC CAT GTG CTT CCT TC
mouse Defb3	5'-GTC TCC ACC TGC AGC TTT TAG
	5'-AGG AAA GGA ACT CCA CAA CTG C
mouse Defb4	5'-ACA ATT GCC AAT CTG TCG AA
	5'-GCA GCC TTT ACC CAA ATT ATC
mouse S100a8	5'-TCA AGA CAT CGT TTG AAA GGA AAT C
	5'-GGT AGA CAT CAA TGA GGT TGC TC
mouse S100a9	5'-AAA GGC TGT GGG AAG TAA TTA AGA G
	5'-GCC ATT GAG TAA GCC ATT CCC
mouse Reg3b	5'-CTC TCC TGC CTG ATG CTC TT
	5'-GTA GGA GCC ATA AGC CTG GG
mouse Gapdh	5'-CGT CCC GTA GAC AAA ATG GT
	5'-TTG ATG GCA ACA ATC TCC AC
mouse Polr2a	5'-CTG GTC CTT CGA ATC CGC ATC
	5'-GCT CGA TAC CCT GCA GGG TCA
mouse Lcn2	Primer Set VMPS-3457, Biomol

Supplementary Table 1. The list of qPCR primers.
